# Dysbiosis of gut microbiota and metabolomic alterations in myasthenia gravis: insights from 16S rRNA sequencing and untargeted metabolomics

**DOI:** 10.3389/fimmu.2026.1799199

**Published:** 2026-04-23

**Authors:** Yunan Shan, Wei Chen, Yanbin Li

**Affiliations:** 1 Department of Neurology, The First Affiliated Hospital of Shandong First Medical University & Shandong Provincial Qianfoshan Hospital, Shandong Institute of Neuroimmunology, Shandong Key Laboratory of Rheumatic Disease and Translational Medicine, Jinan, China; 2 Department of Gastroenterology, Shanghai Sixth People’s Hospital Affiliated to Shanghai Jiao Tong University School of Medicine, Shanghai, China

**Keywords:** 16S rRNA sequencing, biomarkers, butanoic acid, gut microbiota, bile acids, metabolomics, myasthenia gravis, short-chain fatty acids

## Abstract

**Introduction:**

Myasthenia gravis (MG) is an autoimmune disorder of neuromuscular transmission. Gut dysbiosis has been implicated in autoimmune pathogenesis, yet integrated microbial and metabolomic profiling in MG remains scarce. To characterize gut microbiota and the fecal metabolome in MG, identify diagnostic biomarkers, and explore associations between microbial taxa, metabolites, and clinical severity.

**Methods:**

Fecal samples from 29 MG patients and 10 healthy controls underwent 16S rRNA sequencing and UHPLC-Q-TOF MS metabolomics. LEfSe identified differential taxa; metabolites were screened by VIP > 1.0, *P* < 0.05, FDR q < 0.05. Random Forest and Spearman correlation assessed biomarker performance and microbiota – metabolite – clinical associations.

**Results:**

MG patients showed significantly reduced alpha- and beta-diversity. LEfSe identified 232 discriminative taxa, with depletion of *butanoic acid*-producing commensals (*Faecalibacterium prausnitzii, Ruminococcus bromii, Bifidobacterium bifidum*) and enrichment of *Klebsiella*. Metabolomics revealed 567 altered metabolites (424 downregulated), including reduced short-chain fatty acids and secondary bile acids (*lithocholic, isolithocholic*, and *allolithocholic acid*). The Random Forest metabolite model achieved AUC = 1.0. Spearman analysis revealed that *lithocholic acid* (P < 0.05) and *allocholic acid* (P < 0.001) showed positive correlations with the QMGS, while *Ruminococcus* abundance was positively correlated with *butanoic acid* (P < 0.01). KEGG analysis implicated cholinergic synapse, bile secretion, sphingolipid signaling, and mTOR pathways.

**Conclusions:**

MG patients exhibit a distinct profile of gut dysbiosis and metabolic disturbances. The specific microbial and metabolic biomarkers identified in this study may offer novel insights for auxiliary diagnosis of MG and guide future microbiota-targeted intervention strategies.

## Graphical Abstract

**Figure:**
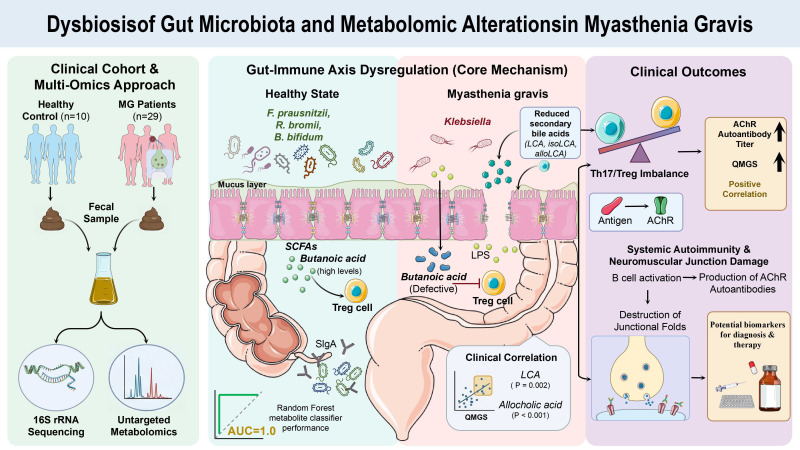

## 1 Introduction

Myasthenia gravis (MG) is an acquired autoimmune disorder driven by both humoral and cellular immune mechanisms. It arises from impaired transmission at the neuromuscular junction (NMJ), manifesting clinically as fatigable weakness of the ocular or skeletal muscles (1, 2). The principal pathogenic antibodies identified in MG target the acetylcholine receptor (AChR), muscle-specific kinase (MuSK), and lipoprotein receptor-related protein 4 (LRP4) (2). The global prevalence of MG is estimated at approximately 173.3 cases per million population (3). Management of MG typically requires long-term immunosuppressive therapy which remains the cornerstone of management, aiming to achieve minimal disease manifestation or sustained clinical improvement (4).

The gut microbiota, a complex symbiotic ecosystem, plays a pivotal role in host physiology by regulating nutrient metabolism, immune homeostasis, and pathogen resistance (5). Emerging evidence highlights its contribution to autoimmune disease (AD) development, with dysbiosis implicated in neuroimmune disorders and systemic ADs, including systemic lupus erythematosus (SLE), multiple sclerosis (MS), inflammatory bowel disease (IBD), and rheumatoid arthritis (RA) (6–9). Certain beneficial species — such as *Faecalibacterium prausnitzii*(*F. prausnitzii)*, *Akkermansia muciniphila(A. muciniphila)*, and *Roseburia* spp. — produce short-chain fatty acids (SCFAs) with potent immunomodulatory properties, promoting regulatory T cell (Treg) differentiation while suppressing pro-inflammatory pathways (9). Conversely, pathogenic species such as *P. copri* and *R. gnavus* compromise intestinal barrier integrity and drive Th17-mediated inflammation, thereby exacerbating immune dysregulation and disease progression (9). Such microbiota – metabolite – immune crosstalk underscores the gut microbiome’s potential as a therapeutic target in autoimmune disorders. Despite advances in understanding MG immunopathology, the contributions of gut dysbiosis and associated metabolomic perturbations to disease pathogenesis remain poorly characterized. To address this gap, we integrated 16S rRNA gene sequencing and untargeted metabolomics to profile fecal samples from MG patients and healthy controls. Our objectives were to define gut microbial and metabolomic signatures in MG, identify potential diagnostic biomarkers, and examine associations between microbial composition and key metabolites. Collectively, this study provides novel insights into the gut microbiota – metabolome axis in MG pathogenesis and proposes candidate biomarkers with diagnostic and therapeutic relevance.

## 2 Methods

### 2.1 Human subjects and sample collection

This study enrolled MG patients who were newly diagnosed or under follow-up at the First Affiliated Hospital of Shandong First Medical University between January and December 2023. Inclusion criteria were: (1) confirmed MG diagnosis according to established diagnostic criteria; (2) age 18–80 years; and (3) provision of written informed consent. Patients were excluded if they met any of the following criteria: (1) use of antibiotics or probiotics within the preceding 3 months; (2) plasma exchange therapy within the preceding 3 months; (3) high-dose intravenous immunoglobulin therapy within the preceding 6 months; (4) pregnancy or lactation; (5) major comorbidities involving the liver, gallbladder, kidney, cardiovascular system, or hematopoietic system, or any concurrent autoimmune disease; (6) chronic gastrointestinal disorders, including irritable bowel syndrome or inflammatory bowel disease; (7) intestinal preparation procedures within the preceding month; (8) malignant thymoma or other malignant neoplasms; or (9) refusal to participate by the patient or their family. Demographic and clinical data were recorded for all participants, including sex, age at onset, thymoma status (confirmed by chest CT and/or pathological examination), AChR and MuSK antibody profiles, MGFA classification, disease status at sampling, and current treatment regimens. Normality of all continuous variables was assessed using the Shapiro-Wilk test. Non-normally distributed data are presented as median (interquartile range). Spearman rank correlation was used to examine the relationship between Quantitative Myasthenia Gravis Score (QMGS) and AChR antibody titers. Sex-based differences in disease severity and antibody levels were assessed using the Mann-Whitney U test.

Healthy controls were recruited from individuals undergoing routine health examinations at the same institution during the same period (January – December 2023). All controls were asymptomatic with no history of neuromuscular or autoimmune disease, and none had presented with suspected MG symptoms subsequently found to be seronegative. Eligibility was confirmed through review of physical examination reports, clinical history, and standard laboratory investigations. Inclusion required: (1) normal physical examination findings with no clinically significant abnormalities; (2) no disorders involving the nervous, digestive, urinary, cardiovascular, or hematopoietic systems; and (3) no significant deviations in routine blood or biochemical parameters. Individuals who had used antibiotics, probiotics, or other microbiota-modulating agents within the preceding three months, or who were pregnant or lactating, were excluded.

The study protocol was approved by the Ethics Committee of the First Affiliated Hospital of Shandong First Medical University (Approval No. YXLL-KY-2023 [075]). Written informed consent was obtained from all participants prior to enrollment.

### 2.2 Fecal specimen collection, DNA extraction, and sequencing

Fecal specimens were collected from participants using a sterilized fecal collection container provided along with a cooler containing ice packs. Immediately after collection, the sample were placed in the cooler and transported to the laboratory, where they were promptly frozen at - 80 °C until further processing for DNA extraction. Total genomic DNA samples were extracted from fecal samples using the OMEGA Soil DNA Kit (M5635-02) (Omega Bio-Tek, Norcross, GA, USA), according to the manufacturer’s instructions. The extracted DNA was stored at -20 °C until subsequent analysis. The quantity and quality of the extracted DNAs were assessed using a NanoDrop NC2000 spectrophotometer (Thermo Fisher Scientific, Waltham, MA, USA) and agarose gel electrophoresis, respectively. The bacterial 16S rRNA genes V3–V4 region was amplified via PCR using the forward primer 338F (5’- ACTCCTACGGGAGGCAGCA-3’) and the reverse primer 806R (5’- GGACTACHVGGGTWTCTAAT-3’). Sample-specific 7-bp barcodes were incorporated into the primers to enable multiplex sequencing.

The PCR amplicons were purified using Vazyme VAHTSTM DNA Clean Beads (Vazyme, Nanjing, China) and quantified using the Quant-iT PicoGreen dsDNA Assay Kit (Invitrogen, Carlsbad, CA, USA). After individual quantification, the amplicons were pooled in equal amounts and subjected to pair-end 2*250 bp sequencing on the Illumina NovaSeq platform with NovaSeq 6000 SP Reagent Kit (500 cycles) (**Supplementary Tables 1**, **2**).

### 2.3 Microbiome bioinformatics analyses

Microbiome bioinformatics analyses were performed conducted using QIIME2 2022.11 (10), with minor modifications based on the official tutorials. Briefly, raw sequence data were demultiplexed using the demux plugin followed by primers cutting with the cutadapt plugin (11). Sequences were then quality filtered, denoised, merged and chimera removed using the DADA2 plugin (12). Non-singleton amplicon sequence variants (ASVs) were aligned with mafft (13) and used to construct a phylogeny with fasttree2 (13). Alpha-diversity metrics (Chao1 (14), Observed species, Shannon, Simpson (15), Faith’s PD (16). Beta diversity metrics (weighted UniFrac (17), and unweighted UniFrac (17) were estimated using the diversity plugin. Taxonomy was assigned to ASVs using the classify-sklearn naïve Bayes taxonomy classifier in the feature-classifier plugin (18)against the SILVA Release 138.

Sequence data analyses were mainly performed using QIIME2 and R packages (v3.2.0). ASV-level alpha diversity indices, such as Chao1 richness estimator, Observed species, Shannon diversity index, Simpson index, and Faith’s PD were calculated using the ASV table in QIIME2 and visualized as box plots. ASV-level ranked abundance curves were generated to compare the richness and evenness of ASVs among samples. Beta diversity analysis was performed to investigate the structural variation of microbial communities across samples using Jaccard metrics, and UniFrac distance metrics and visualized via principal coordinate analysis (PCoA, Bray-Curtis distance) and nonmetric multidimensional scaling (NMDS, weighted uniFrac distance) (17, 19). The significance of differentiation of microbiota structure among groups was assessed by PERMANOVA (Permutational multivariate analysis of variance) (20), ANOSIM(Analysis of similarities) (21, 22), and Permdisp (23) using QIIME2. The taxonomy compositions and abundances were visualized using MEGAN (Huson, Mitra, et al. and GraPhlAn (24). Venn diagram was generated to visualize the shared and unique ASV among samples or groups using the R package “VennDiagram”, based on the occurrence of ASV across samples/groups regardless of their relative abundance (25). Random forest analysis was applied to discriminate the samples from different groups using QIIME2 with default settings (26). Using Linear Discriminant Analysis Effect Size (LEfSe, Kruskal-Wallis and Wilcoxon rank sum test, LDA score > 2.0, *P* < 0.05) analysis, we sought out the robust different species between the two groups, namely the biomarker species. To account for multiple comparisons in taxon-level differential abundance testing, p-values derived from the Kruskal-Wallis test within the LEfSe framework were corrected using the Benjamini-Hochberg (BH) false discovery rate (FDR) method. Taxa were considered significantly differential if they met both criteria, FDR-adjusted q-value < 0.05 and LDA score > 2.0. Adjusted q-values for all tested taxa are reported in **Supplementary Table 3**. The KEGG pathway enrichment analysis method was used to study the metabolic pathways, and the Fisher exact probability test was employed to calculate the significance of the enrichment of different bacterial communities in each pathway, thereby determining the pathways that were significantly affected in MG.

### 2.4 Metabolomics analysis

High-resolution non-targeted metabolomics analysis was performed on fecal samples using UHPLC-Q-TOF MS. The samples were separated on an Agilent 1290 Infinity LC Ultra-High Performance Liquid Chromatography System (UHPLC) with a HILIC column. QC samples were used to ensure the consistency and reliability of the LC-MS assay. The samples were then analyzed by mass spectrometry using a Triple TOF6600 mass spectrometer (AB SCIEX) with electrospray ionization (ESI) in positive and negative ion modes. Metabolite identification was achieved by matching retention time, molecular mass (molecular mass error within <10 ppm), secondary fragmentation spectra, and collision energy of the metabolites in the databases. The results of the identifications were rigorously manually double-checked and confirmed.

The raw data were converted to mzXML format by ProteoWizard and processed with XCMS software for peak alignment, retention time correction, and peak area extraction. The extracted data were used for metabolite structure identification and data preprocessing, followed by data quality evaluation. CAMERA (Collection of Algorithms of MEtabolite pRofile Annotation) was employed for the annotation of isotopes and adducts. Only variables with more than 50% of the nonzero measurement values in at least one group were retained for further analysis. Metabolite identification was performed by comparing of accuracy m/z value (<10 ppm), and MS/MS spectra with an in-house database established with authentic standards.

Differential metabolites between MG and HC groups were identified using a combination of multivariate statistical analysis (OPLS-DA; VIP > 1.0) and univariate analysis (Student’s t-test). To control for the inflated false discovery rate arising from simultaneous testing of thousands of metabolite features, all univariate p-values were corrected using the BH false discovery rate (FDR) method, implemented in R (v4.2.0) using the p.adjust function (method BH). Metabolites were considered significantly differential if they satisfied all of the following criteria: VIP > 1.0, unadjusted p < 0.05, and FDR-adjusted q < 0.05. Both unadjusted p-values and adjusted q-values are reported for all differential metabolites in **Supplementary Table 4**. The results were visualized separately for the positive and negative ion mode. Random Forest analysis was performed on the differential metabolites, and feature selection was performed using the Repeated Feature Elimination (RFE) algorithm. The significance of each metabolite in the Random Forest model was assessed by calculating the classification error (classif.ce).

ROC curves were plotted for each identified differential metabolite, and the area under the curve (AUC) was calculated. AUC values range from 0.5 to 1.0, with AUC > 0.5 indicating a better classification effect. Specifically, AUC values between 0.5 and 0.7 suggest lower accuracy, values between 0.7 and 0.9 indicate moderate accuracy, and values ≥0.9 represent high accuracy. An AUC of 0.5 indicates no diagnostic value. Additionally, enrichment analysis of differential metabolites was performed using Fisher’s Exact Test to calculate the significance level of metabolite enrichment for each pathway. This analysis identified the metabolic and signaling pathways that were significantly affected. Using the PICRUSt2 software, the 16S rRNA gene sequences were predicted in multiple functional databases, including KEGG (https://www.kegg.jp/) and COG (https://www.ncbi.nlm.nih.gov/COG/). A distance matrix was calculated and analyzed using the PCoA analysis method in the R script to output the PCoA coordinates of the sample points and plot them as a two-dimensional scatter plot. The fitFeatureModel function was called, using the zero-corrected log-normal model to fit the distribution of each pathway/group, and the fitting results of this model were used to determine the significance of the differential pathways. To address the high dimensionality of the data and control the false positive rate, all *P* values from univariate analyses (such as Wilcoxon rank sum test and Student’s t-test) were corrected for multiple comparisons using the BH-FDR method. The adjusted *P* value (q value) < 0.05 was considered statistically significant.

### 2.5 Intestinal microbiota-metabolite association analysis

To elucidate the interactions between the gut microbiota and host metabolism, we performed an integrated analysis of 16S rRNA sequencing and metabolomics data. The relative abundance of significantly different microbial taxa (LEfSe LDA > 2, and *P* < 0.05) and the expression of significantly different metabolites (identified by OPLS-DA with VIP > 1, and *P* < 0.05) were compiled into a single table. This table served as the input file for subsequent correlation analyses. The correlation coefficients between the significantly different flora and metabolites identified in the experimental samples were analyzed using Spearman’s rank correlation method. The results were visualized using R language graphing, allowing for a comprehensive exploration of the interactions between the microbiota and metabolites from multiple perspectives. Correlation analysis was conducted between the abundance of key differential bacteria, the levels of key differential metabolites, and clinical indicators (QMGS and acetylcholine receptor antibody titer). To explore whether gut metabolites may mediate the relationship between specific bacteria and clinical outcomes, we applied bootstrap-based mediation analysis using rank-transformed ordinary least-squares regression, an approach suitable for non-normally distributed data with small sample sizes.

## 3 Results

### 3.1 General characteristics

A total of 29 MG patients and 10 healthy controls (HCs) were enrolled. The MG group comprised 11 males and 18 females (mean age 55.38 ± 13.97 years); the HC group comprised 4 males and 6 females (mean age 52.10 ± 11.90 years). Baseline demographic and clinical characteristics — including age, cholesterol and blood glucose levels, and medical histories (e.g., diabetes mellitus, hypertension, coronary heart disease, hepatitis, tuberculosis, prior surgery, and history of blood product transfusion) — were comparable between groups, with no statistically significant differences observed. No significant differences in age, QMGS, or AChR antibody concentration were observed between male and female patients (**Supplementary Table 5**). Spearman rank correlation analysis revealed no significant association between QMG score and serum AChR antibody titer in this cohort (r_s_ = 0.205, *P* = 0.286), suggesting that a single absolute AChR antibody titer is insufficient to independently predict clinical muscle weakness severity.

### 3.2 Differences in gut microbial composition between MG patients and HCs

**Figure 1:**
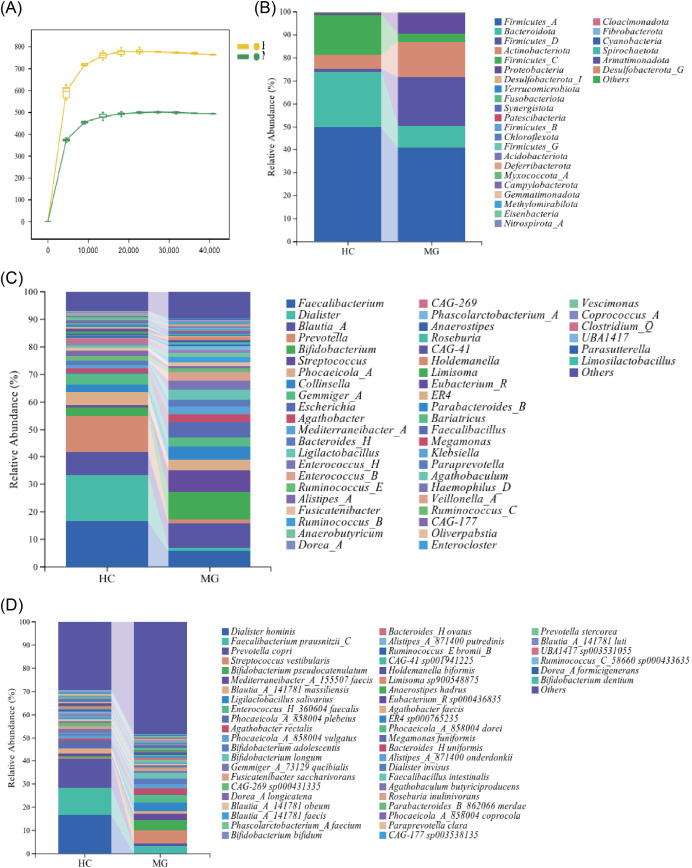
Differences in gut flora between MG and healthy individuals. **(A)** sparse curve (the horizontal axis represents the flattening depth, and the vertical axis shows the median value of the alpha diversity index calculated 10 times along with the box plot); **(B)** Variation in the top 28 abundant phylum between groups; **(C)** Variation in the top 50 abundant genus between groups; **(D)** Variation in the top 50 abundant species between groups.

To evaluate differences in gut bacterial communities between MG patients and HCs, fecal samples were analyzed by 16S rRNA gene sequencing. Following generation of ASV and OTU representative sequences, ASV/OTU tables were normalized and rarefaction curves were constructed. The degree of curvature reflects the extent to which sequencing depth affects the diversity of the observed samples, all curves reached stable plateau levels, confirming that the sequencing depth was sufficient to capture the microbial diversity of each sample (**Figure 1A**). Stacked bar plots were used to visualize bacterial community composition across all taxonomic levels, revealing distinct compositional differences between the MG and HC groups (**Figures 1B–D**).

### 3.3 α and β diversity changes in gut bacteria communities in MG patients

**Figure 2:**
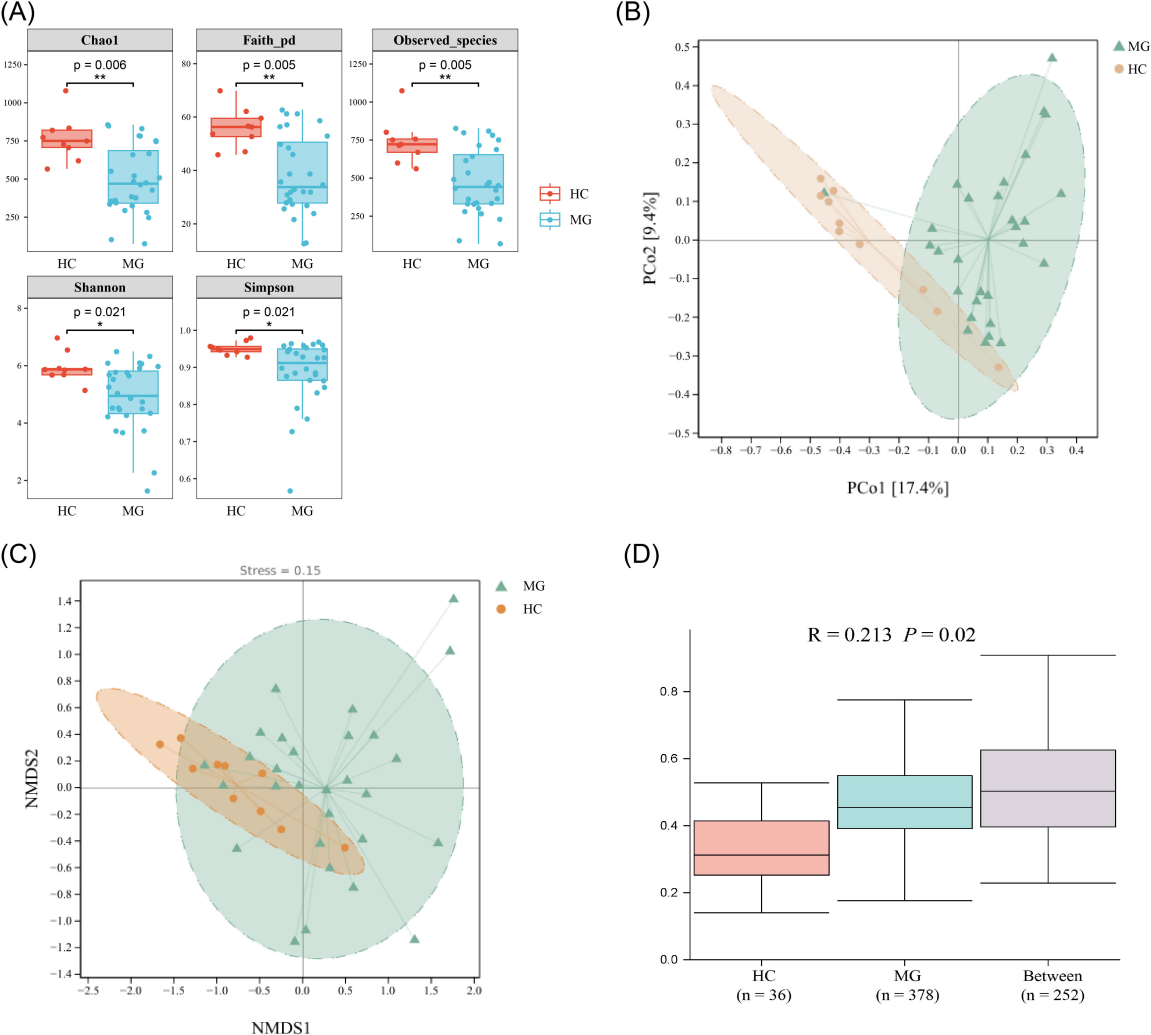
Changes in α and β diversity between HC and MG groups. **(A)** Box plots of differences in alpha diversity between groups **(B)** PCA of the community composition of the groups; **(C)** NMDS analysis reflecting the nonlinear structure of the bacteria community composition of the groups; **(D)** Weighted UniFrac distance box plots for differences in β-diversity between groups. *P < 0.05, **P < 0.01.

Both α-diversity and β-diversity of gut bacterial communities were assessed to comprehensively characterize compositional differences between MG patients and HCs. α-Diversity was significantly lower in the MG group than in HCs across all indices evaluated (**Figure 2A**).

β-Diversity was assessed to examine compositional differences in gut microbial communities between groups. Bray-Curtis dissimilarity matrices were visualized using principal coordinate analysis (PCoA; **Figure 2B**) and non-metric multidimensional scaling (NMDS; **Figure 2C**). Both ordination methods revealed significant separation between the MG and HC groups, with the NMDS stress value of 0.15 confirming adequate model fit. These findings were further supported by weighted UniFrac distance analysis and analysis of similarities (ANOSIM), which demonstrated that between-group differences were significantly greater than within-group variation (*P* = 0.02; **Figure 2D**).

### 3.4 Biomarker analysis and functional prediction between MG patients and healthy controls

**Figure 3:**
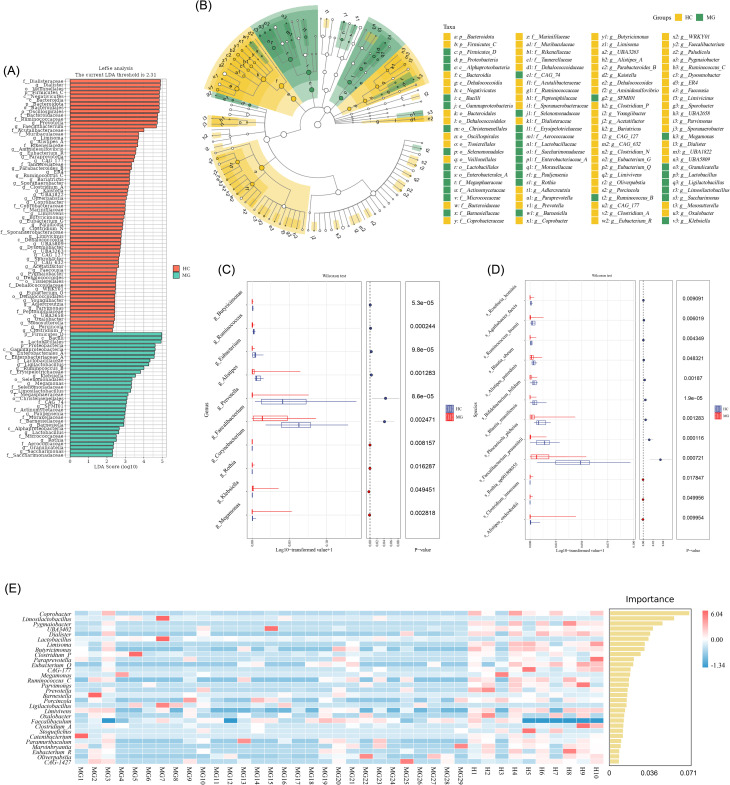
Microbial biomarker analysis between MG and HC. **(A)** LDA histograms of colonies with significant differences between HC and MG groups; **(B)** Taxonomic hierarchical relationships of the major taxonomic units from phylum to genus (inner to outer circle) of differential flora; **(C)** Analysis of differences in differential flora between HC and MG groups at the genus level; **(D)** Analysis of differences in differential flora between HC and MG groups at the species level; **(E)** Randomized forest analysis heatmap of differential flora between HC and MG groups at the genus level.

LEfSe analysis identified 232 taxa with significantly different relative abundances between the MG and HC groups (**Figures 3A, B**). Genus- and species-level differential markers were visualized using box plots based on relative abundance and LDA scores (**Figures 3C, D**). At the family level, *Actinomycetaceae*, *Aerococcaceae*, *Barnesiellaceae*, *Enterobacteriaceae*, *Erysipelotrichaceae*, *Moraxellaceae*, *Mycobacteriaceae*, and *Selenomonadaceae* were significantly enriched in the MG group, while *Rikenellaceae* and *Ruminococcaceae* were significantly depleted (all *P* < 0.05). At the genus level, *Megamonas*, *Klebsiella*, *Rothia*, and *Corynebacterium* were increased, whereas *Faecalibacterium*, *Prevotella*, *Alistipes*, *Eubacterium*, *Ruminococcus*, and *Butyricimonas* were significantly reduced in MG patients (all *P* < 0.05). At the species level, *Alistipes onderdonkii*, *Clostridium innocuum*, and *Rothia* sp001808955 were more abundant, while *F. prausnitzii*, *Phocaeicola plebeius*, *Blautia massiliensis*, *Bifidobacterium bifidum* (*B. bifidum*), *Alistipes putredinis*, *Blautia obeum*, *Ruminococcus bromii* (*R. bromii*), *Agathobacter faecis*, and *Roseburia hominis (R. hominis)* were significantly decreased. These microbial signatures represent potential biomarkers for distinguishing MG patients from healthy individuals. A Random Forest classifier was further constructed based on genus-level marker taxa to discriminate between groups (**Figure 3E**).

**Figure 4:**
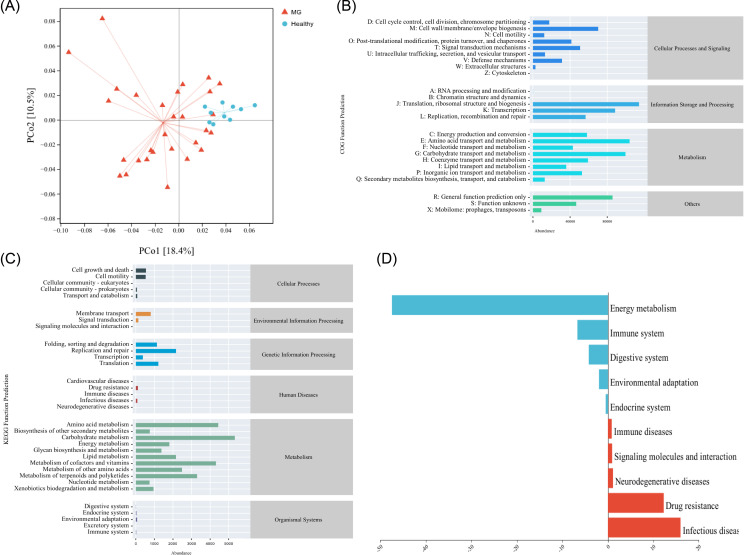
Functional prediction between HC and MG groups. **(A)** Functional unit PCoA analysis for PICRUSt2 analysis; **(B)** Intergroup functional pathway abundance and its classification based on COG database analysis; **(C)** Intergroup functional pathway abundance and its classification based on KEGG database analysis; **(D)** Differences in abundance of differential functional pathways in the MG group based on the COG database.

Species are ranked by descending importance to the classifier, with top-ranked taxa representing the most discriminative markers between groups. Functional differences among samples were visualized by applying principal coordinate analysis (PCoA) to a Jaccard distance matrix (**Figure 4A**). Functional abundance profiles were predicted from 16S rRNA gene sequences using the KEGG database, providing a comprehensive characterization of pathway-level differences between groups (**Figures 4B, C**).

KEGG analysis revealed significant enrichment of pathways associated with immune diseases, infectious diseases, neurodegenerative diseases, and antimicrobial resistance in MG patients, while pathways involved in energy metabolism, endocrine function, digestive physiology, environmental adaptation, and immune regulation were significantly depleted (**Figure 4D**).

### 3.5 Variations in gut metabolomics between MG patients and healthy controls

#### 3.5.1 Differential analysis of significant metabolites

**Figure 5:**
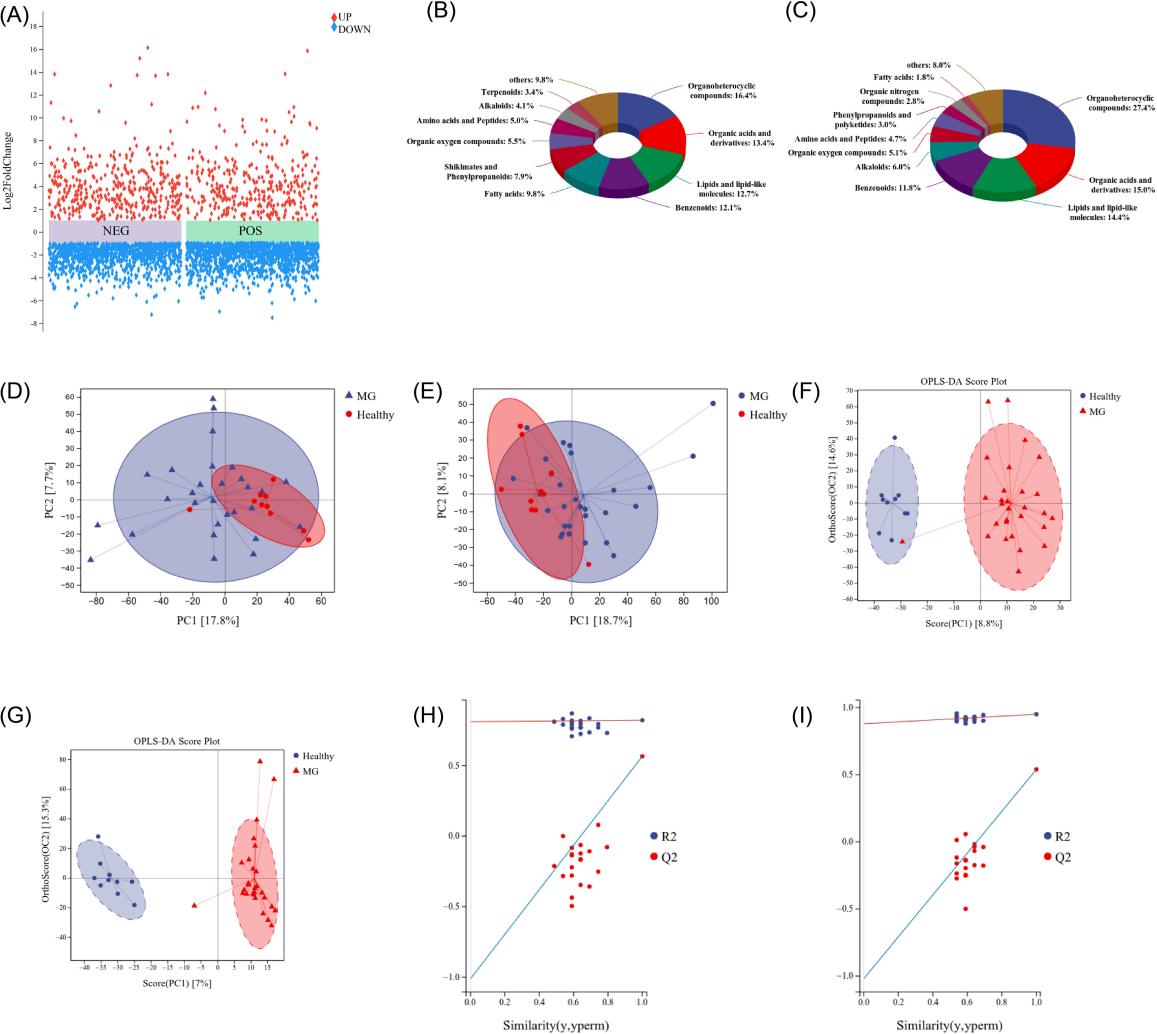
Differential distribution of intestinal metabolites between MG and HC groups. **(A)** Differential metabolite volcano plots between HC and MG groups in positive and negative ion mode, with metabolites satisfying FC > 1 and P value < 0.05 shown in red and metabolites satisfying FC < 1 and *P* < 0.05 shown in blue; **(B)** The chemical classification of all metabolites in the negative ion model is attributed, and the proportion of each metabolite class is shown in a pie chart; **(C)** The chemical classification of all metabolites in the positive ion model is attributed, and the proportion of each metabolite class is shown in a pie chart; **(D)** Positive ion mode PCA score plot (R = 0.528); **(E)** Negative ion mode PCA score plot (R = 0.505); **(F)** Positive ion mode OPLS-DA scoring chart; **(G)** Negative ion mode OPLS-DA scoring chart; **(H)** OPLS-DA permutation test plot in positive ion mode; **(I)** OPLS-DA permutation test plot in negative ion mode.

Untargeted metabolomics was performed using UHPLC-Q-TOF MS, identifying a total of 3,341 metabolites (1,154 in positive ion mode and 2,187 in negative ion mode). Differential metabolites were screened by combined univariate and multivariate statistical analyses. Fold change (FC) analysis was applied to all detected metabolites, with those reaching *P* < 0.05 visualized in volcano plots (**Figure 5A**). Metabolites were categorized according to chemical taxonomy, and the proportions of positive and negative ion metabolites are presented in pie charts (**Figures 5B, C**). Principal component analysis (PCA) was used to examine group separation of positive and negative ion metabolite profiles (**Figures 5D, E**). Orthogonal partial least squares discriminant analysis (OPLS-DA) was subsequently applied to further resolve between-group differences, with model performance evaluated using R²X, R²Y, Q², and OPLS-DA score plots (**Figures 5F–I**).

**Figure 6:**
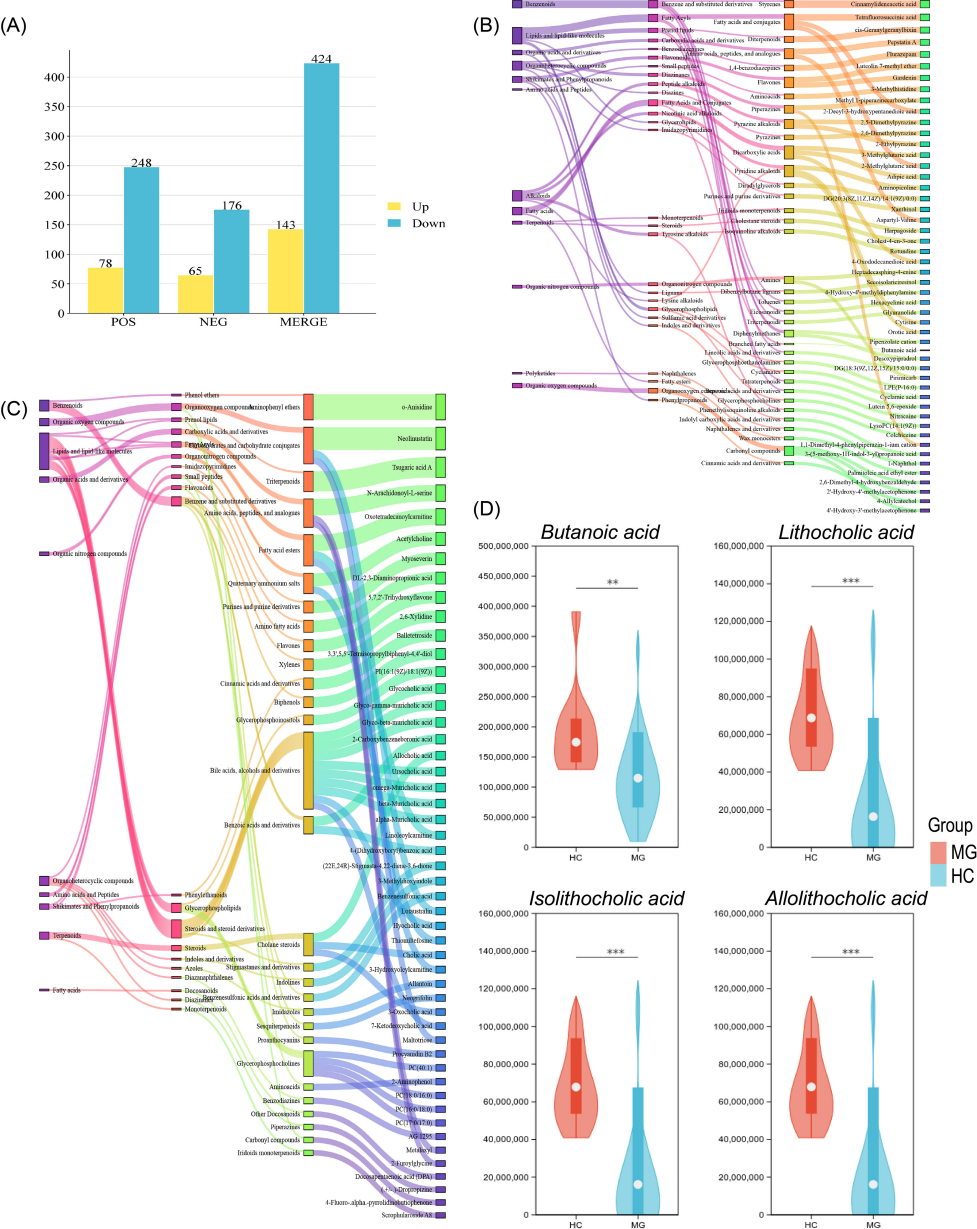
Analysis of intestinal differential metabolites between MG and HC groups. **(A)** Significantly different metabolites with VIP > 1 and *P* < 0.05 obtained from OPLS-DA analysis; **(B)** Top 50 metabolites with elevated difference values in MG and their attribution; **(C)** Top 50 metabolites with reduced difference values in MG and their attribution; **(D)** The differences in the contents of *butanoic acid*, *LCA*, *isoLCA* and *alloLCA acid* between HC and MG. **P < 0.01, ***P < 0.005.

A total of 567 significantly differential metabolites were identified between MG patients and HCs (VIP > 1.0, *P* < 0.05, FDR-corrected q < 0.05), of which 143 were upregulated and 424 were downregulated in the MG group. These metabolites were categorized by ion mode and are presented as bar graphs (**Figure 6A**). Pearson’s correlation coefficients were calculated for all pairwise metabolite comparisons to assess the consistency of directional changes, with results visualized as a correlation heatmap (**Figure 6B**).

Further analysis identified the top five upregulated and downregulated metabolites in both ion modes. In negative ion mode, the most upregulated metabolites were *7-Chloro-5-(1H-pyrrol-2-yl)-1,3-dihydro-1,4-benzodiazepin-2-one*, *(E)-1-Hydroxy-2-methylbut-2-enyl-4-diphosphate*, *Balletetroside*, *Glycocholic acid* and *2-Carboxybenzeneboronic acid*. while the most downregulated metabolites were *Cinnamylideneacetic acid*, *Tetrafluorosuccinic acid*, *Luteolin 7-methyl ether*, *2-Decyl-3-hydroxypentanedioic acid* and *3-Methylglutaric acid*. In positive ion mode, the top upregulated metabolites were *o-Anisidine*, *Neolinustatin*, *Tsugaric acid A*, *N-Arachidonoyl-L-serine*, and *Oxotetradecanoylcarnitine*, whereas the most downregulated metabolites were cis- *Geranylgeranylbixin*, *Pepstatin A*, *Flurazepam*, *3-[(5,6-Diphenylfuro[2,3-d]pyrimidin-4-yl)amino]-1-propanol*, and *Gardenin*. Sankey diagrams were used to illustrate the attributed subgroups of upregulated and downregulated metabolites in the MG group (**Figures 6B, C**). Among the 567 statistically significant differential metabolites, short-chain fatty acids (SCFAs) emerged as a particularly prominent class of dysregulated compounds. *Butanoic acid (butyrate)* was significantly reduced in MG patients compared to healthy controls (p = 0.005; FDR q = 0.02; VIP = 1.13), with an area under the receiver operating characteristic curve (AUC) of 0.931, identifying it as the single metabolite with the strongest individual diagnostic performance in this cohort. This is perfectly corroborated by the fact that the abundance of core *butanoic acid*-producing bacteria in the gut of the MG group (represented by Faecalibacterium) significantly decreased, suggesting a coordinated loss of microbial community-dependent short-chain fatty acid biosynthesis capacity in MG. In addition, the levels of secondary bile acids (such as *LCA*, *IsoLCA*, and *AlloLCA*) and short-chain fatty acids (such as *butanoic acid*) in patients with severe myasthenia gravis (MG) were all reduced. The “violin plot” was used to show the differences in different characteristic metabolites (**Figure 6D**).

Additionally, we compared the differential metabolites in the MG and HC group and constructed the ROC curves, identifying 59 metabolites with an AUC >0.9 (**Supplementary Table 6**).

#### 3.5.2 Changes in metabolic pathways and functions in MG

**Figure 7:**
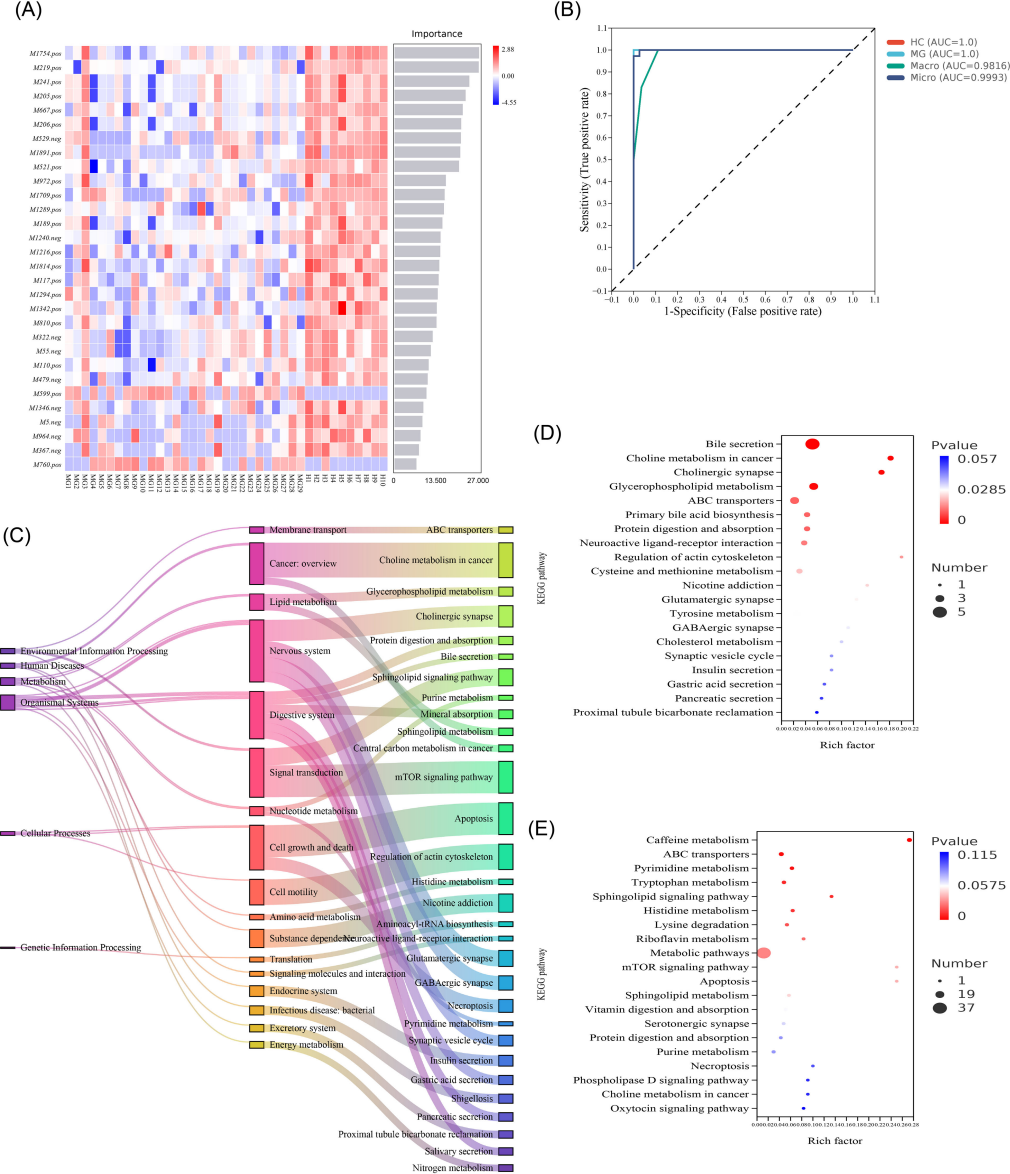
Changes in differential metabolic pathways and functions between MG and HC groups. **(A)** Random Forest plot of differential metabolites between HC and MG groups; **(B)** Randomized forest plot of ROC curves for differential metabolites between HC and MG groups (AUC = 1.0); **(C)** Differential metabolite enrichment analysis between HC and MG groups KEGG pathway hierarchical attribution; **(D)** Enrichment analysis pathway bubble plots for elevated differential metabolites in the MG group; **(E)** Enrichment analysis pathway bubble plots for reduced differential metabolites in the MG group.

The relative importance of differential metabolites was assessed using a Random Forest algorithm, with abundance patterns across MG and HC groups visualized in a heatmap (**Figure 7A**). Model performance was evaluated by ROC curve analysis, demonstrating high discriminative accuracy (AUC = 1.0; **Figure 7B**).

KEGG enrichment analysis quantified pathway enrichment using the rich factor, FDR value, and number of enriched metabolites. The 30 most significantly enriched pathways (P-value ranked) are presented in a Sankey diagram, revealing alterations in pathways related to metabolism, membrane translocation, cell signaling, and cell cycle regulation (**Figure 7C**). KEGG pathway analysis was performed separately for upregulated and downregulated metabolites in the MG group. The top 20 pathways ranked by FDR value are displayed in bubble plots (**Figures 7D, E**). Pathway hierarchy analysis identified significant alterations in purine metabolism, the cholinergic synapse, bile secretion, sphingolipid signaling, and mTOR signaling in MG patients. Collectively, these findings reveal substantial metabolic and pathway-level dysregulation in MG relative to HCs.

### 3.6 Associations between gut microbiota, key metabolites, and clinical indicators in MG

**Figure 8:**
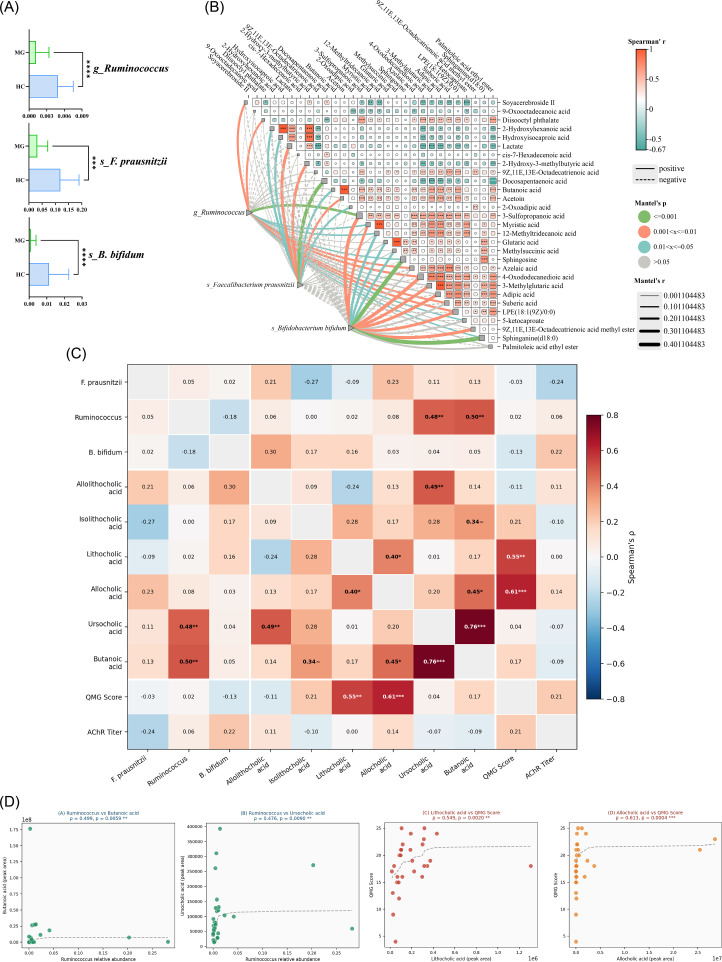
Association analysis of intestinal differential metabolites with differential flora between MG and HC groups. **(A)** Box plots of three differential flora; **(B)** Interactive Mantel test correlation heat map for correlation analysis of three differential bacteria with differential fatty acids. **(C)** Heatmap of Spearman’s rank correlations among the top differential gut microbiota (genus level), key targeted metabolites (including SCFAs and bile acids), and clinical indices (QMGS and AChR antibody titer) within the MG cohort. Color intensity reflects the magnitude of ρ; **(D)** Scatter plots of four key significant correlations: *Ruminococcus* vs *butanoic acid* (ρ = 0.499, *P* = 0.006); *Ruminococcus* vs *ursocholic acid* (ρ = 0.476, *P* = 0.009); *LCA* vs QMGS (ρ = 0.549, *P* = 0.002); *Allocholic acid* vs QMGS (ρ = 0.613, p < 0.001). Dashed lines indicate rank-based trend lines. (~ *P* < 0.1, *P < 0.05, **P < 0.01, ***P < 0.001, ****P < 0.001.

Three bacteria, *Ruminococcus, F. prausnitzii*, and *B. bifidum*, were selected based on abundance and log2FC values, and their abundance differences were visualized (**Figure 8A**). Spearman analysis and Mantel test were used to generate correlation heatmaps, reflecting interactions between these bacteria and 29 differential fatty acids, as well as correlation among significantly differential fatty acids-fatty acids and colonies (**Figure 8B**). The analysis revealed significant associations between these bacteria and fatty acids such as Suberic acid and *butanoic acid*. The finding not only highlight alterations in the gut microbiota and metabolites of MG patients but also provide value insights for identifying potential therapeutic targets and diagnostic markers for MG.

To further explore the clinical relevance of gut microbiota and metabolic alterations in MG, we performed Spearman’s rank correlation analysis linking the abundance of three key bacteria—*F. prausnitzii*, *Ruminococcus* spp., and *B. bifidum*—to the levels of key differential metabolites (secondary bile acids and short-chain fatty acids) and two clinical indicators (QMGS and AChR antibody titer) across all 29 MG patients (**Figure 8C**). The secondary bile acids *allolithocholic acid (alloLCA)*, *isolithocholic acid (isoLCA)*, and *Lithocholic acid* (*LCA)* were all significantly downregulated in MG (all FDR < 0.01), while *ursocholic acid* and *allocholic acid* were markedly elevated (all FDR < 0.001). *Butanoic acid* was also significantly reduced in the MG group (*P* < 0.05).

Spearman’s correlation analysis revealed several statistically significant associations (**Figures 8C, D**). *Ruminococcus* abundance was positively correlated with *butanoic acid* levels (ρ = 0.499, *P* = 0.006) and *ursocholic acid* levels (ρ = 0.476, *P* = 0.009), indicating that the depletion of *Ruminococcus* in MG may co-occur with reduced SCFA and altered secondary bile acid production (**Figure 8B**). Furthermore, *ursocholic acid* abundance was strongly positively correlated with *butanoic acid* (ρ = 0.763, *P* < 0.001), suggesting a co-regulatory relationship between primary bile acid metabolism and *butanoic acid* production. Importantly, *LCA* was positively correlated with QMGS (ρ = 0.549, *P* = 0.002), and *allocholic acid* showed an even stronger positive correlation with QMGS (ρ = 0.613, *P* < 0.001) (**Figures 8C, D**), indicating that elevated bile acid species are associated with greater disease severity. Neither *F. prausnitzii* nor *B. bifidum* reached statistically significant direct correlations with QMGS score or AChR titer, which may reflect the modest sample size and the high clinical heterogeneity of the cohort.

The mediation analysis revealed that for the *Ruminococcus* → *butanoic acid* → QMGS pathway, the indirect effect was 0.106 (95% CI: −0.140 to 0.401), which did not reach statistical significance (*P* = 0.354), likely due to the modest sample size and the near-zero total effect of *Ruminococcus* on QMGS (c = 0.017). Nonetheless, path a (*Ruminococcus* → *butanoic acid*: β = 0.499, *P* = 0.006) was independently robust and statistically significant, supporting the biological plausibility of this axis(**Supplementary Figure 1A**). Similarly, for *F. prausnitzii* → *allocholic acid* → QMGS, the indirect effect of 0.153 (95% CI: −0.082 to 0.376, *P* = 0.205) showed a directionally consistent trend, with *allocholic acid* strongly predicting QMGS (path b: β = 0.653, *P* < 0.001) (**Supplementary Figure 1B**). Taken together, these findings suggest a plausible “microbiota – metabolite – clinical severity” axis, though formal mediation significance was not established in this exploratory cohort, and larger studies are required for confirmation.

## 4 Discussion

Gut microbiota dysbiosis has been identified as a shared feature across multiple autoimmune diseases, including rheumatoid arthritis, multiple sclerosis, inflammatory bowel disease, and MG (1, 6–9). In the present study, integrated 16S rRNA sequencing and UHPLC-Q-TOF MS untargeted metabolomics were applied to fecal samples from 29 MG patients and 10 HCs, enabling simultaneous characterization of microbial composition and metabolite profiles. Our findings reveal coordinated alterations in microbial community structure, SCFA-producing taxa, and secondary bile acid metabolism, converging on a dysbiosis – metabolome axis with implications for MG pathophysiology. Candidate biomarkers and therapeutic targets identified herein warrant validation in larger prospective cohorts. Both α- and β-diversity were significantly reduced in the MG group relative to HCs, consistent with prior observations (27). Reduced microbial richness is broadly associated with impaired gut barrier integrity and immune dysregulation, suggesting that ecosystem-level collapse, rather than disruption of any single taxon, may underlie MG pathogenesis (28).

LEfSe analysis identified 232 discriminative taxa between MG patients and HCs. The most prominently depleted genus was *Faecalibacterium*, whose sole species *F. prausnitzii* ranks among the most abundant *butanoic acid* producers in the healthy human gut (6). Depletion of *F. prausnitzii* has been documented across multiple autoimmune diseases, including SLE, RA, and MS (9). Mechanistically, *butanoic acid* produced by *F. prausnitzii* inhibits histone deacetylases 1 and 3 (HDAC1/3), promoting Foxp3 expression and Treg differentiation while suppressing the IL-6/STAT3/IL-17 axis (7, 29, 30). *Ruminococcaceae* was also markedly reduced, consistent with its central role in SCFA production and its established depletion in Crohn’s disease and type 1 diabetes (31). At the species level, *R. bromii* and *Roseburia hominis*, key degraders of resistant starch that channel carbon flux toward *butanoic acid*, were significantly diminished (32, 33).

*B. bifidum* contributes to gut immune homeostasis through multiple mechanisms, including modulation of dendritic cell maturation and cytokine production, promotion of regulatory immune phenotypes, enhancement of intestinal IgA secretion, and production of exopolysaccharides and short-chain organic acids that maintain mucus layer integrity and colonization resistance (34). Its depletion in MG patients is particularly noteworthy given its capacity to generate estrogen-deconjugating β-glucuronidase enzymes as part of the estrobolome — the ensemble of gut bacterial genes encoding enzymes responsible for metabolizing conjugated estrogens within the intestinal lumen. Dysbiosis-associated loss of estrobolome-competent taxa, including *B. bifidum*, *Lactobacillaceae*, and *Ruminococcaceae*, may reduce enterohepatic estrogen recycling and alter systemic estrogen bioavailability, with potential consequences for hormone – immune interactions that partly govern MG susceptibility and severity, particularly in female patients (35, 36).

Collectively, the concurrent depletion of these three functional guilds — SCFA producers, resistant-starch fermenters, and early-life immunomodulators — represents a coherent collapse of anti-inflammatory microbial capacity in MG patients. Conversely, *Klebsiella*, *Megamonas*, *Rothia*, and *Corynebacterium* were significantly enriched. The expansion of *Klebsiella* is of particular immunological interest. Gram-negative *Klebsiella* species disrupt intestinal barrier integrity via lipopolysaccharide (LPS) shedding and type VI secretion system-mediated displacement of commensal bacteria (37). Moreover, antigenic peptides of *Klebsiella* share structural homology with host self-antigens, providing a potential molecular mimicry route for autoantibody cross-reactivity (38, 39). Whether analogous cross-reactivity exists between *Klebsiella* epitopes and AChR subunits warrants direct investigation in future experimental autoimmune MG (EAMG) models.

PICRUSt2 functional prediction revealed enrichment of pathways associated with immune disorders, infectious diseases, and neurodegenerative processes in MG patients, alongside significant deficits in energy metabolism, lipid biosynthesis, and vitamin synthesis. These functional shifts likely reflect the metabolic consequences of depleting fermentative commensals and are consistent with multi-omics findings from other autoimmune cohorts (9, 40).

Untargeted metabolomics identified 567 significantly altered metabolites (VIP > 1.0, *P* < 0.05, FDR-corrected *q* < 0.05), of which 424 were downregulated and 143 upregulated in the MG group. The Random Forest model achieved AUC = 1.0, underscoring the strong discriminative capacity of the fecal metabolome. Among individual metabolites, *butanoic acid* was significantly reduced (*P* < 0.05; AUC = 0.931), a finding mechanistically consistent with the concurrent depletion of *butanoic acid*-producing taxa identified by 16S rRNA sequencing. In AChR-positive MG patients, *butanoic acid* deficiency has been shown to impair Treg differentiation through dysregulation of mTOR-mediated autophagy: *butanoic acid* inhibits mTOR signaling, thereby activating autophagy, increasing CTLA-4 surface expression, and restoring Treg suppressive function (41). Concordantly, *butanoic acid* supplementation has been shown to reduce anti-AChR antibody titers and clinical scores in EAMG mouse models (41, 42). Of particular relevance to NMJ physiology is the emerging evidence that SCFAs, and *butanoic acid* in particular, exert direct and indirect effects on peripheral neuromuscular function that extend well beyond their established roles in intestinal homeostasis (43). At the level of the enteric nervous system (ENS), *butanoic acid* serves as the primary energy substrate for colonocytes and modulates enteric neuronal function through histone deacetylase (HDAC) inhibition and GPR41/GPR43 receptor signaling (44). Critically, *butanoic acid* has been demonstrated to enhance the integrity of the intestinal epithelial barrier by upregulating tight junction proteins (occludin, claudin-1, ZO-1), thereby limiting the translocation of microbial products (including lipopolysaccharide (LPS) from enriched gram-negative taxa such as *Klebsiella*) into the systemic circulation (45). In MG, where NMJ function is already compromised by pathogenic autoantibodies, this *butanoic acid*-deficiency-driven increase in intestinal permeability may provide a chronic source of systemic microbial antigen exposure that sustains the dysregulated immune environment contributing to disease persistence and exacerbation (46, 47). These findings position gut-derived *butanoic acid* as a promising endogenous immunomodulator in MG. However, current evidence remains largely derived from *in vitro* and rodent studies, and clinical translation requires validation in well-designed human trials.

Secondary bile acids exhibited a complex, bidirectional pattern of dysregulation. *LCA, alloLCA*, and *isoLCA* were markedly downregulated (FDR < 0.01), while *ursocholic acid* and *allocholic acid* were significantly elevated (FDR < 0.001). *LCA* and its congeners function as endogenous TGR5 agonists, suppressing NF-κB-driven inflammation via cAMP–PKA signaling in dendritic cells and macrophages, and acting as RORγt antagonists to restrain Th17 cell differentiation (19, 48). Their depletion therefore removes a key immunomodulatory brake, consistent with the hyperactivated Th17 state characteristic of MG (6). Spearman correlation analysis revealed that *LCA* abundance positively correlated with QMG score (ρ = 0.549, *P* = 0.002), and *allocholic acid* demonstrated an even stronger association (ρ = 0.613, *P* < 0.001), indicating that bile acid dysregulation tracks with clinical disease severity. *Ruminococcu*s abundance correlated with both *butanoic acid* (ρ = 0.499, *P* = 0.006) and *ursocholic acid* (ρ = 0.476, *P* = 0.009), while ursocholic acid and *butanoic acid* were strongly inter-correlated (ρ = 0.763, *P* < 0.001), suggesting a co-regulatory relationship between bile acid metabolism and SCFA production partly mediated by *Ruminococcus*. Although mediation analyses did not reach formal significance — likely reflecting the modest sample size — the independently significant path coefficients (*Ruminococcus* → *butanoic acid*: β = 0.499, *P* = 0.006; *allocholic acid* → QMGS: β = 0.653, *P* < 0.001) support the biological plausibility of a microbiota – metabolite – clinical severity axis.

Notably, each of these metabolic perturbations is mechanistically traceable to the compositional dysbiosis identified by 16S rRNA sequencing, establishing biological consistency across the microbiome and metabolomics datasets and strengthening confidence in the validity of these findings. The metabolomics-based Random Forest model achieved AUC = 1.0, and *butanoic acid* demonstrated individual biomarker performance of AUC = 0.931, collectively highlighting the translational potential of the fecal metabolome as a source of MG-associated diagnostic biomarkers. These performance metrics, however, were derived from a small discovery cohort and require validation in larger, independent samples before clinical utility can be established.

Key metabolic alterations clustered around five interconnected pathways: SCFA metabolism, secondary bile acid synthesis, amino acid–AhR signaling, Cholinergic synapse and sphingolipid remodeling — all converging on Th17/Treg immune balance (49–53). Beyond their canonical roles in intestinal lipid absorption and hepatic metabolism, secondary bile acids function as potent immunomodulatory signaling molecules acting through two principal receptor systems: the nuclear farnesoid X receptor (FXR) and the membrane-bound G protein-coupled receptor TGR5. FXR activation suppresses hepatic and intestinal inflammation and inhibits NF-κB signaling; critically, it also suppresses production of B cell-activating factor (BAFF) — a cytokine that promotes B cell survival and IgG class switching (49). Impaired FXR signaling resulting from secondary bile acid depletion may therefore contribute to elevated BAFF-driven autoreactive B cell survival and AChR IgG autoantibody production in MG. TGR5 activation by secondary bile acids promotes macrophage M2 polarization, suppresses pro-inflammatory cytokine release, and stimulates enteroendocrine L cells to secrete glucagon-like peptide-1 (GLP-1) (48, 49, 54). GLP-1 has recently been identified as having direct neuroprotective and neuromodulatory effects at the NMJ, GLP-1 receptor (GLP-1R) signaling in motor neurons promotes axonal survival, enhances mitochondrial function, and may modulate synaptic vesicle cycling at the NMJ (55, 56).

Tryptophan-derived indole metabolites — including indole-3-aldehyde (IAld), indole-3-acetic acid (IAA), and indole-3-propionic acid (IPA) — were significantly depleted in the MG metabolome. These indole derivatives are generated through microbiota-dependent catabolism of dietary tryptophan by taxa including Faecalibacterium, Ruminococcaceae, and Lactobacillaceae — all of which were depleted in our MG cohort, establishing directional consistency between the microbiome and metabolomics findings (53). Indole metabolites function as endogenous AhR ligands; AhR activation in intestinal epithelial cells and intraepithelial lymphocytes drives IL-22 production, which maintains tight junction protein expression, stimulates mucin secretion, and promotes intestinal epithelial repair (53). AhR activation also promotes the differentiation of inducible Tregs (iTregs) and suppresses Th17 responses in the intestinal mucosa (53). Depletion of AhR-activating indole metabolites in MG patients therefore impairs both intestinal barrier integrity and AhR-dependent mucosal immunosuppression — two complementary mechanisms that collectively increase systemic exposure to gut-derived microbial antigens and pro-inflammatory signals. Sphingolipids are essential structural and signaling components of neuronal membranes and myelin sheaths. The ceramide/S1P ratio governs a fundamental cell fate balance: ceramide promotes apoptosis, senescence, and inflammatory signaling, whereas S1P promotes cell survival, proliferation, and lymphocyte trafficking via sphingosine-1-phosphate receptor 1 (S1PR1) (57). The gut microbiome regulates host sphingolipid metabolism through multiple mechanisms, including microbial sphingolipid biosynthesis and modulation of host sphingolipid-metabolizing enzymes. Dysbiosis-associated perturbations in sphingolipid profiles have been documented in neurological conditions including multiple sclerosis and Alzheimer’s disease (57).

At the core of MG pathogenesis lies the aberrant formation of pathogenic autoantibodies. Predominantly, these include IgG1/IgG3 anti-AChR antibodies, which disrupt neuromuscular transmission through complement activation, antigenic modulation, and direct receptor blockade; and IgG4 anti-MuSK antibodies, which primarily impair synaptic structural integrity by inhibiting the MuSK-LRP4 interaction (1, 58, 59). The gut microbiota and metabolomic alterations identified in our study are mechanistically positioned as upstream drivers of this autoantibody-mediated pathological cascade, operating through several interconnected immunological pathways (60).

The profound depletion of *butanoic acid*-producing taxa and consequent reduction in intestinal *butanoic acid* observed in our MG cohort is mechanistically central to autoantibody formation. *Butanoic acid*-deficient conditions compromise Foxp3+ Treg induction and follicular regulatory T (Tfr) cell differentiation, thereby impairing the regulatory checkpoints that constrain Tfh-mediated B cell activation in germinal centers (61, 62). The depletion of *butanoic acid* thus creates a permissive immunological milieu in which AChR-specific autoreactive B cells can escape peripheral tolerance, enter germinal center reactions, and undergo somatic hypermutation to generate high-affinity pathogenic IgG1/IgG3 anti-AChR antibodies — the proximate mediators of NMJ destruction in AChR-MG (63, 64). Additionally, the expansion of pro-inflammatory taxa (notably *Klebsiella*) and the concurrent disruption of gut barrier integrity may initiate AChR-specific autoimmunity through molecular mimicry mechanisms, whereby bacterial antigens with structural homology to AChR epitopes activate cross-reactive lymphocytes in the context of a dysbiosis-driven inflammatory environment (61).

A notable finding in our clinical characterization was the absence of a significant correlation between QMGS and AChR antibody titer (*r_s_* = 0.205, *P* = 0.286). This is consistent with the well-established dissociation between antibody titer and functional disease severity in MG, confirming that a single absolute titer is insufficient to independently predict clinical muscle weakness (1, 2). This finding further reinforces the value of multi-omics biomarkers — particularly fecal bile acids — as complementary severity indicators capable of capturing disease activity dimensions not reflected by serology alone.

Several limitations of this study warrant acknowledgment. The sample size of 29 MG patients and 10 HCs reflects its exploratory, pilot nature; while adequate for hypothesis generation, it limits statistical power for subgroup analyses stratified by MGFA classification, antibody subtype, or individual immunosuppressant regimen. Confounding by treatment is inherent in a real-world cohort: prior or concurrent immunosuppression (corticosteroids, azathioprine, mycophenolate mofetil) and thymectomy can independently reshape the gut microbiota and T cell repertoire, and their specific contributions cannot be fully disentangled at this sample size. Biological sex is among the most influential determinants of gut microbiota composition, mediated through bidirectional interactions between sex hormones and the gut microbial ecosystem (35). No significant differences in QMGS or AChR antibody titer were observed between male and female patients (all *P* > 0.77, Mann-Whitney U test), and PERMANOVA with sex as a covariate revealed no significant effect on overall microbial community structure. These results suggest that sex-related confounding does not drive the primary dysbiosis signal, though statistical power to detect sex-stratified effects remains limited. Dysbiosis-associated depletion of estrobolome-competent taxa — including Bifidobacterium, Lactobacillaceae, and Ruminococcaceae, all significantly reduced in our MG cohort — may impair estrogen deconjugation capacity, reduce circulating estrogen bioavailability, and perturb the estrogen – immune axis governing B cell activation and AChR autoantibody production (36). Future studies should prospectively stratify participants by sex and menopausal status, systematically measure circulating estradiol, FSH, and LH, and apply functional metagenomic profiling of the estrobolome to isolate MG-specific dysbiosis signatures from sex hormone-driven microbiota variation (35, 36, 65, 66).

There are several important sources of biological heterogeneity and potential confounding factors in this study, which require explicit discussion. Regarding the confounding factors related to treatment, the MG cohort includes patients with newly diagnosed and untreated cases, as well as those receiving immunosuppressive therapy (corticosteroids, azathioprine, mycophenolate mofetil). Immunosuppressive drugs have been proven to directly alter the composition of the intestinal microbiota by regulating secretory immunoglobulin A in the intestine, changing intestinal peristalsis, and influencing the way the mucosal immune surveillance is carried out (67, 68). We acknowledge that the observed microbiome and metabolomics changes in MG patients are likely to represent the combined signals resulting from the interaction between the intrinsic intestinal flora dysbiosis and treatment-related flora disturbances within MG, and these factors cannot be completely separated in the current cross-sectional study cohort.

Additionally, 16S rRNA sequencing provides genus-to-species-level resolution but cannot capture strain-level variation or direct functional activity; future investigations employing metagenomics and metatranscriptomics would substantially add mechanistic depth. Replication in larger, independent, multi-center cohorts with rigorous stratification by treatment status and clinical subtype, to construct a rigorous “microbiota - metabolites - clinical phenotype” network, is essential before these findings can inform clinical practice. In addition, future studies should ideally recruit and analyze treatment-naïve MG patients as a distinct cohort to establish an unconfounded baseline microbiota signature for MG, and subsequently examine the longitudinal impact of specific therapeutic interventions on gut microbiota evolution. Such a prospective, treatment-stratified design would enable a more rigorous delineation of disease-intrinsic versus treatment-induced microbiota changes in MG.

In summary, this study provides an integrated multi-omics characterization of gut dysbiosis in MG. Concurrent depletion of *butanoic acid*-producing commensals and dysregulation of secondary bile acid metabolism — together with pathobiont expansion, notably *Klebsiella* — exert convergent pressure on Th17/Treg homeostasis and may sustain systemic autoimmune activation. The significant positive correlations between bile acid species and QMG scores delineate a tractable microbiota – metabolite – clinical severity axis. Collectively, these findings highlight the gut microbiome – metabolome interface as a promising target for biomarker development and microbiota-modulating therapeutic strategies in MG, and provide a foundation for future prospective, mechanistic investigation.

## 5 Conclusion

This study provides an integrated multi-omics characterization of gut microbiota dysbiosis and metabolomic alterations in MG, revealing a coherent microbiome – metabolome axis with implications for disease pathogenesis, biomarker development, and therapeutic intervention.

At the microbial level, MG patients exhibited significantly reduced α- and β-diversity alongside depletion of key *butanoic acid*-producing commensals — *F. prausnitzii, R. bromii*, and *B. bifidum* — and concurrent enrichment of pro-inflammatory pathobionts including *Klebsiella*. At the metabolite level, 567 significantly altered metabolites were identified, with prominent reductions in short-chain fatty acids (particularly *butanoic acid*, AUC = 0.931) and secondary bile acids (*LCA*, *isoLCA, alloLCA*). A Random Forest metabolite classifier discriminated MG from healthy controls with AUC = 1.0, underscoring the strong diagnostic potential of the fecal metabolome.

Mechanistically, the depletion of *butanoic acid* impairs mTOR-mediated autophagy and Treg differentiation, while secondary bile acid dysregulation disrupts FXR–BAFF and TGR5 signaling, collectively shifting immune balance toward Th17 hyperactivation. Depletion of tryptophan-derived indole metabolites further compromises AhR-dependent mucosal immunosuppression and intestinal barrier integrity. These interconnected pathway disruptions converge on Th17/Treg imbalance and may collectively drive MG pathogenesis.

Critically, *LCA* (ρ = 0.549, *P* = 0.002) and *allocholic acid* (ρ = 0.613, P < 0.001) positively correlated with QMGS, establishing fecal bile acids as severity-correlated biomarkers that capture disease activity dimensions not reflected by AChR antibody titer alone. These findings highlight the gut microbiome – metabolome interface as a promising target for auxiliary diagnosis and microbiota-directed therapeutic strategies in MG. Validation in larger, prospective, multi-center cohorts is warranted.

## Data Availability

The datasets presented in this study can be found in online repositories. The 16S rRNA gene sequencing raw data have been deposited in the NCBI Sequence Read Archive (SRA) database under the accession number SRP684690. The untargeted metabolomics data have been deposited in the MetaboLights database under the accession number MTBLS14266.
