# Supplementary material for: Dysbiosis of gut microbiota and metabolomic alterations in myasthenia gravis: insights from 16S rRNA sequencing and untargeted metabolomics
Source: Front Immunol. 2026 Apr 23;17:1799199. doi: 10.3389/fimmu.2026.1799199 (PMC13149435; doi:10.3389/fimmu.2026.1799199)
Supplement: Supplementary file 1 [file Table1.docx]

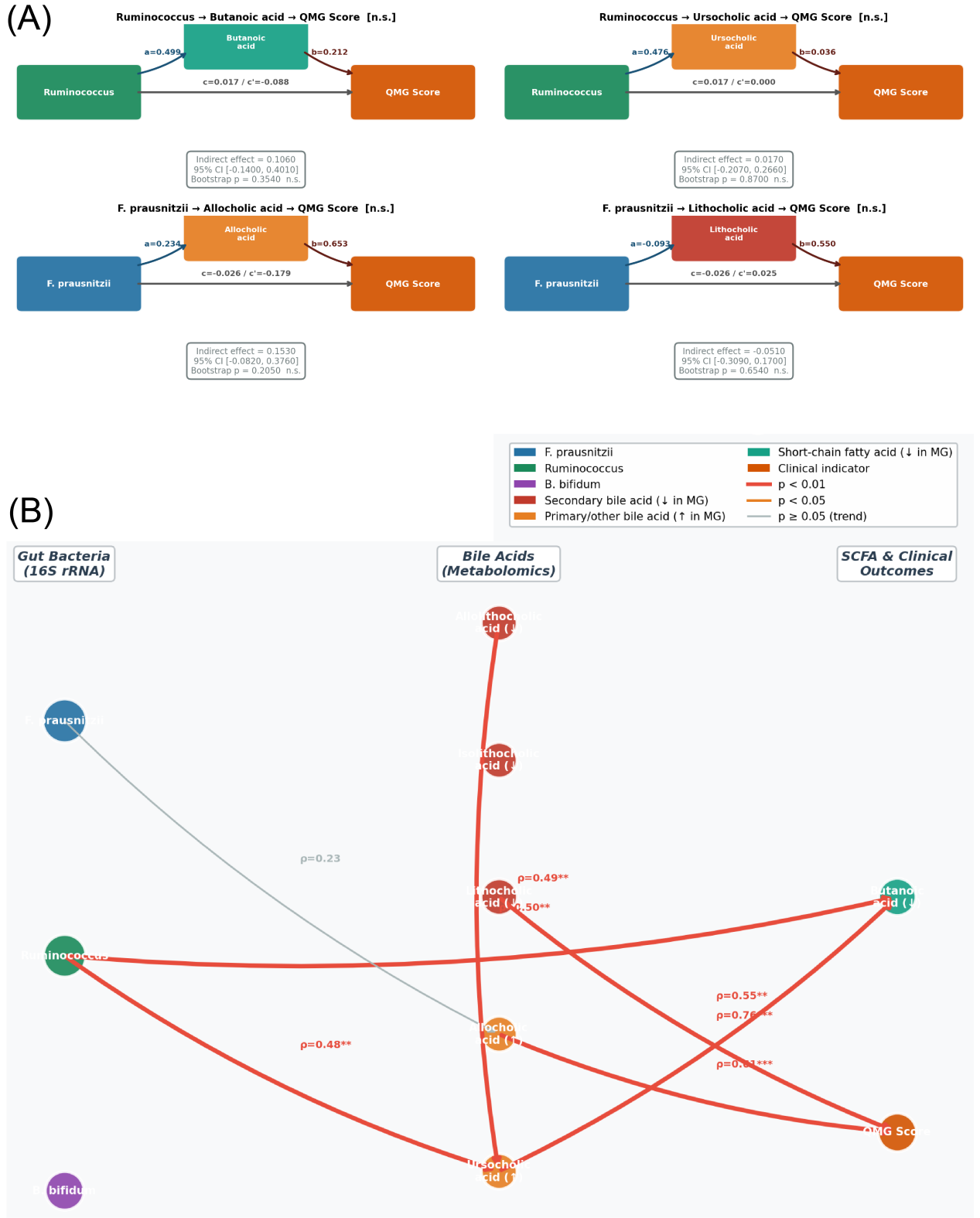


(A) Bootstrap-based mediation path diagrams exploring whether gut metabolites mediate the association between specific bacteria and QMG score. Path a: bacteria → metabolite; path b: metabolite → QMG (controlling for bacteria); c: total effect; c’: direct effect. Indirect effect and 95% CI are shown; n.s. = not significant.

(B) Integrated microbiota–metabolite–clinical phenotype network illustrating Spearman correlations among key nodes. Edge width and color reflect the strength and significance of correlations (red: p < 0.01; orange: p < 0.05; gray: p ≥ 0.05). Arrows indicate direction of association. Node colors denote biological category.
