# Supplementary material for: Dysbiosis of gut microbiota and metabolomic alterations in myasthenia gravis: insights from 16S rRNA sequencing and untargeted metabolomics
Source: Front Immunol. 2026 Apr 23;17:1799199. doi: 10.3389/fimmu.2026.1799199 (PMC13149435; doi:10.3389/fimmu.2026.1799199)
Supplement: Supplementary file 2 [file Table2.docx]

**Supplementary Table.1 PCR Run System**

| **components** | **volumes (μL)** |
| --- | --- |
| Q5 high-fidelity DNA polymerase | 0.25 |
| 5*Reaction Buffer | 5 |
| 5* High GC Buffer | 5 |
| dNTP（10mM） | 2 |
| DNA Template | 2 |
| Forward primer（10uM） | 1 |
| Reverse primer（10uM） | 1 |
| ddH_2_O | 8.75 |
