# Supplementary material for: Dysbiosis of gut microbiota and metabolomic alterations in myasthenia gravis: insights from 16S rRNA sequencing and untargeted metabolomics
Source: Front Immunol. 2026 Apr 23;17:1799199. doi: 10.3389/fimmu.2026.1799199 (PMC13149435; doi:10.3389/fimmu.2026.1799199)
Supplement: Supplementary file 3 [file Table3.docx]

**Supplementary Table.2 PCR Runtime Program**

| Programs | Time |
| --- | --- |
| 98℃ | 5min |
| 98℃ | 30s |
| 53℃ | 30s (25 cycles) |
| 72℃ | 45s |
| 72℃ | 5min |
| 12℃ | ∞ |
