# Supplementary material for: Dysbiosis of gut microbiota and metabolomic alterations in myasthenia gravis: insights from 16S rRNA sequencing and untargeted metabolomics
Source: Front Immunol. 2026 Apr 23;17:1799199. doi: 10.3389/fimmu.2026.1799199 (PMC13149435; doi:10.3389/fimmu.2026.1799199)
Supplement: Supplementary file 4 [file Table4.docx]

**Significant differential metabolites between the MG and HC groups**

| ***Genus*** | **Group** | **LDA_score** | **P-value** | **q-value (FDR)** |
| --- | --- | --- | --- | --- |
| *Coprobacter* | HC | 2.99649 | 3.12E-07 | 1.20E-05 |
| *Pygmaiobacter* | HC | 2.51475 | 3.86E-07 | 1.20E-05 |
| *Limisoma* | HC | 3.72034 | 5.06E-07 | 1.20E-05 |
| *Limosilactobacillus* | MG | 3.29107 | 7.84E-06 | 0.000114894 |
| *CAG_177* | HC | 3.45082 | 9.59E-06 | 0.000114894 |
| *Catenibacterium* | HC | 2.25308 | 9.71E-06 | 0.000114894 |
| *Parvimonas* | HC | 2.42956 | 1.23E-05 | 0.000124499 |
| *Dialister* | HC | 4.90726 | 2.10E-05 | 0.000186058 |
| *Lactobacillus* | MG | 2.64422 | 3.43E-05 | 0.000270735 |
| *Mesosutterella* | HC | 2.37137 | 4.79E-05 | 0.000316871 |
| *Butyricimonas* | HC | 2.93186 | 4.91E-05 | 0.000316871 |
| *Prevotella* | HC | 4.73639 | 7.99E-05 | 0.000430767 |
| *Eubacterium_Q* | HC | 2.44945 | 8.75E-05 | 0.000430767 |
| *UBA3402* | HC | 2.29409 | 8.77E-05 | 0.000430767 |
| *Eubacterium_R* | HC | 3.56798 | 9.10E-05 | 0.000430767 |
| *Paraprevotella* | HC | 3.5099 | 0.000131679 | 0.000584326 |
| *Sporobacter* | HC | 2.64462 | 0.000176807 | 0.000738427 |
| *Ruminococcus_C* | HC | 3.23191 | 0.00022656 | 0.000837747 |
| *Faecalicoccus* | HC | 2.16262 | 0.000235985 | 0.000837747 |
| *CAG_632* | HC | 2.62814 | 0.000235985 | 0.000837747 |
| *CAG_194* | HC | 2.22699 | 0.000272909 | 0.000880751 |
| *UBA1822* | HC | 3.09786 | 0.000272909 | 0.000880751 |
| *Limivivens* | HC | 2.96324 | 0.000390356 | 0.00120501 |
| *Peptococcus* | HC | 2.28951 | 0.000548649 | 0.00162309 |
| *Paramuribaculum* | HC | 2.19873 | 0.000801677 | 0.00227676 |
| *Alistipes_A* | HC | 3.68063 | 0.00120572 | 0.00319299 |
| *Clostridium_P* | HC | 2.30762 | 0.00121424 | 0.00319299 |
| *Marvinbryantia* | HC | 2.05798 | 0.00149679 | 0.00374476 |
| *Duodenibacillus* | HC | 2.0568 | 0.00158138 | 0.00374476 |
| *CAG_127* | HC | 2.69009 | 0.00163553 | 0.00374476 |
| *Dehalococcoides* | HC | 2.49263 | 0.00168778 | 0.00374476 |
| *WRKY01* | HC | 2.46151 | 0.00168778 | 0.00374476 |
| *Limivicinus* | HC | 2.7786 | 0.00175561 | 0.00377723 |
| *Oliverpabstia* | HC | 3.05743 | 0.00184505 | 0.00385291 |
| *Faecalibacterium* | HC | 4.68944 | 0.00233031 | 0.00472719 |
| *Megamonas* | MG | 3.3355 | 0.00263958 | 0.00520584 |
| *Barnesiella* | MG | 2.91794 | 0.00381848 | 0.00724734 |
| *Dysosmobacter* | HC | 2.73105 | 0.00396126 | 0.00724734 |
| *Odoribacter* | HC | 2.24294 | 0.00398093 | 0.00724734 |
| *Ruminococcus_B* | MG | 4.00965 | 0.00418376 | 0.00742618 |
| *Porcincola* | HC | 2.34494 | 0.00460033 | 0.00796642 |
| *Bariatricus* | HC | 3.23154 | 0.0051437 | 0.00869531 |
| *SFMI01* | MG | 3.03614 | 0.00755173 | 0.012326 |
| *Corynebacterium* | MG | 2.20015 | 0.00770934 | 0.012326 |
| *Ligilactobacillus* | MG | 4.27157 | 0.00793152 | 0.012326 |
| *Acetatifactor* | HC | 2.62643 | 0.00798584 | 0.012326 |
| *UBA3263* | HC | 2.71055 | 0.0104439 | 0.0150465 |
| *Clostridium_N* | HC | 2.84843 | 0.0104439 | 0.0150465 |
| *Faecalibaculum* | MG | 2.2011 | 0.0105496 | 0.0150465 |
| *Youngiibacter* | HC | 2.43107 | 0.0114438 | 0.0150465 |
| *UBA5809* | HC | 2.75881 | 0.0114438 | 0.0150465 |
| *Kaistella* | HC | 3.13685 | 0.0114438 | 0.0150465 |
| *Aminidesulfovibrio* | HC | 3.57993 | 0.0114438 | 0.0150465 |
| *Sporanaerobacter* | HC | 3.22337 | 0.0114438 | 0.0150465 |
| *CAG_1427* | HC | 2.15474 | 0.0132547 | 0.0171106 |
| *Oxalobacter* | HC | 2.41716 | 0.0141799 | 0.0179781 |
| *Rothia* | MG | 2.50552 | 0.015476 | 0.0192771 |
| *ER4* | HC | 3.25136 | 0.0159241 | 0.0194933 |
| *Paludicola* | HC | 2.86546 | 0.018089 | 0.0217681 |
| *CAG_83* | HC | 2.23442 | 0.0209936 | 0.0248424 |
| *CAG_317* | HC | 2.23328 | 0.0220812 | 0.0257011 |
| *Adlercreutzia* | HC | 2.42986 | 0.0228219 | 0.0257704 |
| *Clostridium_A* | HC | 3.17126 | 0.0228667 | 0.0257704 |
| *Pauljensenia* | MG | 2.97983 | 0.0279931 | 0.0310549 |
| *Granulicatella* | MG | 2.33959 | 0.0305009 | 0.0333164 |
| *UBA2658* | HC | 2.423 | 0.0389872 | 0.0419408 |
| *Saccharimonas* | MG | 2.30971 | 0.0416672 | 0.0441548 |
| *Faecousia* | HC | 2.53959 | 0.0442907 | 0.0462447 |
| *Parabacteroides_B* | HC | 3.33587 | 0.0467022 | 0.0480559 |
| *Klebsiella* | MG | 3.5456 | 0.0474369 | 0.0481146 |
| *Eubacterium_G* | HC | 2.8798 | 0.049665 | 0.049665 |
