# Supplementary material for: Dysbiosis of gut microbiota and metabolomic alterations in myasthenia gravis: insights from 16S rRNA sequencing and untargeted metabolomics
Source: Front Immunol. 2026 Apr 23;17:1799199. doi: 10.3389/fimmu.2026.1799199 (PMC13149435; doi:10.3389/fimmu.2026.1799199)
Supplement: Supplementary file 5 [file Table5.docx]

**Identification of key differential metabolites between the MG and HC groups following FDR correction**

| **Name** | **foldChange** | **log2FoldChange** | **pvalue** | **FDR** | **vip** | **type** | **Regulation** |
| --- | --- | --- | --- | --- | --- | --- | --- |
| Cyclamic acid | 0.221825467 | -2.172503086 | 0.013446793 | 0.048406693 | 1.149878928 | NEG | Down |
| N-Benzyladenine | 0.483031201 | -1.049811712 | 0.006421055 | 0.026103586 | 1.234057344 | POS | Down |
| N2-Benzyl-1,3,5-triazine-2,4-diamine | 0.76777222 | -0.381249734 | 0.035269737 | 0.092982347 | 1.04355325 | POS | Down |
| 3-[(Pyridin-3-ylmethyl)amino]-1H-pyrazole-4-carboxamide | 0.390979494 | -1.354835151 | 0.005902064 | 0.024652638 | 1.556694711 | POS | Down |
| 7-Hydroxy-1,3-dimethyl-2,4-dioxo-1,2,3,4-tetrahydro-6-pteridinecarbaldehyde | 0.426229804 | -1.230296617 | 0.019892581 | 0.065127765 | 1.484670759 | NEG | Down |
| 3-Methylcyclohexanethiol | 0.418412932 | -1.257000653 | 0.000665866 | 0.004928634 | 1.163188631 | POS | Down |
| Aspartyl-Valine | 0.134849717 | -2.890575596 | 0.014099952 | 0.047291661 | 1.379521432 | POS | Down |
| 7H-Pyrrolo[3,2-f]quinazoline-1,3-diamine | 0.819134273 | -0.287828136 | 0.023862865 | 0.074361773 | 1.045156326 | NEG | Down |
| 17-Trifluoromethylphenyl-13,14-dihydrotrinorprostaglandin F1.alpha. | 0.075681237 | -3.723920512 | 5.66E-05 | 0.000886411 | 1.995213389 | NEG | Down |
| Gardenin | 0.093841709 | -3.413626906 | 0.039038341 | 0.098925305 | 1.29819891 | POS | Down |
| Carmoxirole | 0.640403071 | -0.642947868 | 0.008291695 | 0.034090837 | 1.066584038 | NEG | Down |
| 4-(7-Hydroxy-3-(hydroxymethyl)-5-(3-hydroxypropyl)-2,3-dihydro-1-benzofuran-2-yl)-2-methoxyphenyl .beta.-D-xylopyranoside | 0.497427829 | -1.007440872 | 0.000493381 | 0.004271044 | 1.376278413 | NEG | Down |
| 5-VALEROLACTONE | 0.335722333 | -1.574659584 | 0.002873061 | 0.014709126 | 1.471701235 | POS | Down |
| 3,7-Dimethyluric acid | 0.617878688 | -0.694604483 | 8.80E-07 | 5.04E-05 | 2.096128556 | NEG | Down |
| 16:0 PE (1,2-dipalmitoyl-sn-glycero-3-phosphoethanolamine) | 0.208835384 | -2.25956192 | 0.017665802 | 0.055662633 | 1.051806146 | POS | Down |
| Benzene, 1-fluoro-2-[(1E)-2-(4-methoxyphenyl)ethenyl]- | 0.7417103 | -0.431072291 | 0.005637675 | 0.023760285 | 1.006499209 | POS | Down |
| Luteolin 7-methyl ether | 0.081922304 | -3.609599894 | 0.003085135 | 0.016306719 | 1.687354152 | NEG | Down |
| 2-(3-Nitrophenyl)-4-quinolinecarboxylic acid | 0.574314362 | -0.800087454 | 0.00280718 | 0.015278285 | 1.108023428 | NEG | Down |
| Vigabatrin | 0.346076346 | -1.530837755 | 0.000555175 | 0.004666357 | 1.259781464 | NEG | Down |
| 3,6-Dichloro-5-nitro-4-pyridazinamine | 0.438268352 | -1.190113592 | 0.01685109 | 0.053850638 | 1.178566019 | POS | Down |
| 2,4,6-Trimethoxypyrimidine | 0.589919185 | -0.761410767 | 0.002639528 | 0.013735821 | 1.288697256 | POS | Down |
| Palmitoleic acid ethyl ester | 0.241529522 | -2.049728557 | 0.000890798 | 0.006155597 | 1.250183735 | POS | Down |
| 4-[3-Methyl-5-(5,6,7-trihydroxy-1,2,4a,5-tetramethyl-3,4,6,7,8,8a-hexahydro-2H-naphthalen-1-yl)pentoxy]-4-oxobutanoic acid | 0.27780073 | -1.847877706 | 0.010488617 | 0.040746237 | 1.541136524 | NEG | Down |
| 4-Allyl-3-(4-chlorobenzyl)-1H-1,2,4-triazole-5-thione | 0.728663996 | -0.456674388 | 0.005492551 | 0.025308091 | 1.077266602 | NEG | Down |
| Anserine | 0.317424315 | -1.655515452 | 0.000165019 | 0.001765865 | 1.545293704 | POS | Down |
| Rosmaricine | 0.556107609 | -0.846564017 | 0.001803693 | 0.010330508 | 1.117394847 | POS | Down |
| 2-Oxo-S-phenyl-2-(piperidin-1-yl)ethane-1-sulfonamido | 0.63457303 | -0.656141889 | 0.002907994 | 0.01564114 | 1.144319318 | NEG | Down |
| N-(2,3-Dimethylphenyl)-4-methylbenzenesulfonamide | 0.539059082 | -0.89148469 | 0.000660711 | 0.005273296 | 1.578590834 | NEG | Down |
| Salmoxanthin | 0.553787878 | -0.85259462 | 0.000438593 | 0.003634238 | 1.437762387 | POS | Down |
| Glu-Thr | 0.627680151 | -0.671898507 | 0.022286623 | 0.070773868 | 1.093778863 | NEG | Down |
| 2,8,9-Triisobutyl-2,5,8,9-tetraaza-1-phosphabicyclo[3.3.3]undecane solution | 0.006793285 | -7.201674864 | 5.68E-05 | 0.000794087 | 2.679326512 | POS | Down |
| (2-Biphenyl)dicyclohexylphosphine | 0.614907783 | -0.701558028 | 0.027846545 | 0.077870527 | 1.003109609 | POS | Down |
| Mardepodect | 0.495115918 | -1.014161761 | 0.049855507 | 0.118430544 | 1.080501807 | POS | Down |
| 3-[(5,6-Diphenylfuro[2,3-d]pyrimidin-4-yl)amino]-1-propanol | 0.087914991 | -3.507747007 | 0.000150749 | 0.001634962 | 2.469577377 | POS | Down |
| Nefazodone | 0.839723461 | -0.2520138 | 0.003219178 | 0.015976451 | 1.026410716 | POS | Down |
| Galactosyl_4-hydroxyproline | 0.509914577 | -0.971672513 | 0.001030334 | 0.006857339 | 1.173828774 | POS | Down |
| Nervobscurine | 0.510215321 | -0.970821873 | 0.004459466 | 0.020123115 | 1.112280362 | POS | Down |
| Cryptotanshinone | 0.279613553 | -1.838493802 | 5.82E-06 | 0.00016932 | 1.717658694 | POS | Down |
| (2,5-Dioxotetrahydrofuran-3-yl)acetic acid | 0.284489596 | -1.813552203 | 2.67E-05 | 0.000525145 | 1.991309477 | NEG | Down |
| 3-Methylhistidine | 0.102654968 | -3.284124653 | 0.000529136 | 0.004198711 | 2.191835324 | POS | Down |
| 3,12-Dihydroxy-13-methoxypodocarpa-8,11,13-trien-7-one | 0.347081382 | -1.526654117 | 5.89E-05 | 0.00080733 | 1.551143175 | POS | Down |
| Gly-Pro-Arg | 0.311588227 | -1.682287372 | 0.01180678 | 0.044271399 | 1.63897199 | NEG | Down |
| Glycylproline | 0.429982748 | -1.21764932 | 2.15E-05 | 0.000396375 | 2.012768033 | POS | Down |
| delta-Tocotrienol | 0.449065829 | -1.155001148 | 0.006736333 | 0.027089991 | 1.317739729 | POS | Down |
| Celereoin | 0.562688119 | -0.829592593 | 0.00027166 | 0.002556426 | 1.324467846 | POS | Down |
| L-Pyridosine | 0.697004861 | -0.520759378 | 0.001719885 | 0.010002395 | 1.217386324 | POS | Down |
| Lys-Arg | 0.249993128 | -2.000039655 | 0.001024757 | 0.007240473 | 1.666865564 | NEG | Down |
| Polyporusterone_A | 0.265531236 | -1.913046514 | 0.001451121 | 0.008988311 | 1.425106889 | POS | Down |
| 2,4-Dodecadienamide, N-(p-hydroxyphenethyl)-, (E,E)- | 0.617381847 | -0.695765031 | 0.028574226 | 0.085134157 | 1.019960177 | NEG | Down |
| 5-(4-Aminophenyl)-4-phenyl-2,4-dihydro-3H-1,2,4-triazole-3-thione | 0.425170229 | -1.233887513 | 2.44E-05 | 0.000494466 | 1.542298128 | NEG | Down |
| 4-Hydroxy-3-[1-(5-hydroxy-2,6,6-trimethyltetrahydro-2H-pyran-2-yl)ethyl]-2(1H)-quinolinone | 0.071846413 | -3.798940057 | 0.016198362 | 0.052481198 | 1.519161192 | POS | Down |
| Lys-Tyr | 0.33942035 | -1.558855032 | 0.005103023 | 0.02210392 | 1.154536931 | POS | Down |
| Stercobilin | 0.249281669 | -2.004151299 | 1.38E-05 | 0.000295793 | 1.628066743 | POS | Down |
| 3'-Hydroxystanozolol | 0.636547501 | -0.651659919 | 0.003895055 | 0.018373146 | 1.229518252 | POS | Down |
| Ethanone, 2-(4-methylphenyl)-1-(1-pentyl-1H-indol-3-yl)- | 0.370902172 | -1.430889377 | 3.65E-06 | 0.000123028 | 1.75893872 | POS | Down |
| Serpentine cation | 0.259634148 | -1.945447948 | 7.19E-06 | 0.000188876 | 1.754848501 | POS | Down |
| N-Cyclohexylbenzenecarbothioamide | 0.662058899 | -0.594968524 | 0.001744534 | 0.010102176 | 1.145531287 | POS | Down |
| Tri(butoxyethyl)phosphate | 0.391183014 | -1.354084367 | 0.01737537 | 0.055180795 | 1.093231661 | POS | Down |
| Pipenzolate cation | 0.209178035 | -2.257196728 | 0.009040422 | 0.033949926 | 1.215002312 | POS | Down |
| Urocanic acid | 0.561047704 | -0.83380465 | 0.016798504 | 0.05728833 | 1.218225027 | NEG | Down |
| 1H-Indazole-1-pentanoic acid, 3-[[[1-(aminocarbonyl)-2,2-dimethylpropyl]amino]carbonyl]- | 0.150676069 | -2.73047779 | 6.34E-05 | 0.000962753 | 1.862302812 | NEG | Down |
| 5-Chloro-2-(4-propionyl-1-piperazinyl)aniline | 0.574052288 | -0.800745942 | 0.001946773 | 0.011025171 | 1.158561554 | POS | Down |
| 4-[(2,2-Diphenylacetyl)oxy]-1,1-dimethylpiperidinium cation | 0.177957247 | -2.490397412 | 6.89E-06 | 0.00018791 | 2.144810434 | POS | Down |
| 1-Piperazineacetic acid, 4-(phenylmethyl)-, 2-[[2-hydroxy-3-(2-propen-1-yl)phenyl]methylene]hydrazide | 0.082815063 | -3.593962985 | 0.001334528 | 0.00838633 | 2.165350594 | POS | Down |
| 8-Hydroxy-3-(7-hydroxyheptyl)-6-oxo-3,4-dihydroisochromene-7-carboxylic acid | 0.311996652 | -1.680397546 | 0.02530568 | 0.077973836 | 1.09026826 | NEG | Down |
| 4-(7-Hydroxy-6-methoxy-2,3-dimethyl-1,2,3,4-tetrahydronaphthalen-1-yl)benzene-1,2-diol | 0.595897287 | -0.746864415 | 0.001792186 | 0.010934124 | 1.365318136 | NEG | Down |
| atrazine | 0.440281977 | -1.183500305 | 0.000431961 | 0.003584572 | 1.718551566 | POS | Down |
| Calcipotriol | 0.470431167 | -1.087944449 | 0.003170523 | 0.015830921 | 1.039781644 | POS | Down |
| Pirimicarb | 0.218743224 | -2.192689771 | 0.045997822 | 0.111434138 | 1.634476628 | POS | Down |
| 4-Hydroxycyclofenil | 0.829904137 | -0.268983396 | 0.001894637 | 0.011419761 | 1.335448025 | NEG | Down |
| 2-(4-Methyl-5-thiazolyl)ethyl_isobutyrate | 0.682532483 | -0.551030387 | 0.000521628 | 0.004156748 | 1.292582956 | POS | Down |
| 3-(1,1-Dimethyl-2-propenyl)-8-(3-methyl-2-butenyl)xanthyletin | 0.102468002 | -3.286754634 | 0.04400493 | 0.107828702 | 1.320408923 | POS | Down |
| Heptadecasphing-4-enine | 0.162000648 | -2.625928514 | 0.000501151 | 0.004033616 | 1.824626229 | POS | Down |
| DG(20:3(8Z,11Z,14Z)/14:1(9Z)/0:0) | 0.123708599 | -3.014982314 | 8.39E-05 | 0.001044918 | 1.855364122 | POS | Down |
| 8-Hydroxycarvedilol | 0.320300909 | -1.642500201 | 0.000596516 | 0.004541124 | 1.35162148 | POS | Down |
| Dehydropipernonaline | 0.184737418 | -2.436451988 | 0.004070406 | 0.018918176 | 1.405583042 | POS | Down |
| Riboflavin | 0.567508682 | -0.817285632 | 0.004769735 | 0.021049781 | 1.045170843 | POS | Down |
| Cyclopiazonic acid | 0.878519992 | -0.186852978 | 0.031410698 | 0.091502176 | 1.163033743 | NEG | Down |
| Propyphenazone | 0.585560443 | -0.772109997 | 0.021591201 | 0.064418145 | 1.117408592 | POS | Down |
| LPE(18:1(9Z)/0:0) | 0.454630138 | -1.137234768 | 5.35E-06 | 0.000160863 | 1.688979807 | POS | Down |
| 6,7,8,9,14,15-Hexahydro-7-methyl-5H-indolo[3,2-f][3]benzazecine | 0.321753844 | -1.635970712 | 0.000281911 | 0.002762323 | 1.37557756 | NEG | Down |
| 4-Hydroxy-4'-methyldiphenylamine | 0.172939586 | -2.531659957 | 0.000192824 | 0.001991328 | 2.160143584 | POS | Down |
| Madurastatin B2 | 0.439736031 | -1.185290347 | 0.000220404 | 0.00230435 | 1.849212208 | NEG | Down |
| 2-Methoxy-3-methyl-9H-carbazole | 0.478212196 | -1.064277171 | 0.021450126 | 0.064065288 | 1.401241976 | POS | Down |
| 5-ketocaproate | 0.417737474 | -1.259331527 | 0.003265161 | 0.017077632 | 1.115782072 | NEG | Down |
| 4'-Chloro-2-hydroxy-4-methoxybenzophenone | 0.273576472 | -1.869983931 | 5.66E-05 | 0.000886411 | 1.920398954 | NEG | Down |
| 1,2,3,4-Tetrahydroisoquinoline-6-carboxylic acid | 0.208940372 | -2.258836817 | 0.000458852 | 0.00376326 | 1.551767586 | POS | Down |
| 7-Hydroxy-5-methylflavone | 0.353522845 | -1.500124648 | 0.002137784 | 0.012422197 | 1.72102631 | NEG | Down |
| 2-(4-Methoxyphenyl)quinazolin-4-ol | 0.398277233 | -1.328155083 | 0.001030459 | 0.007262095 | 1.448186542 | NEG | Down |
| 2-Butylquinoline | 0.539924411 | -0.88917065 | 0.012250551 | 0.042562519 | 1.168400995 | POS | Down |
| 7-Aminonimetazepam | 0.503126798 | -0.991006061 | 0.004696317 | 0.020847599 | 1.149141279 | POS | Down |
| DG(18:3(9Z,12Z,15Z)/15:0/0:0) | 0.21746246 | -2.20116172 | 0.00329441 | 0.016134653 | 1.352575466 | POS | Down |
| 1-Naphthol | 0.241413039 | -2.050424498 | 3.82E-06 | 0.000148452 | 1.72715941 | NEG | Down |
| Butalbital | 0.315240449 | -1.665475434 | 0.00024933 | 0.002398525 | 2.00565106 | POS | Down |
| [8]-Gingerdione | 0.364905719 | -1.454404333 | 6.83E-05 | 0.000889998 | 1.54006415 | POS | Down |
| Sinapic acid | 0.511995314 | -0.965797488 | 0.048448996 | 0.116021508 | 1.492362643 | POS | Down |
| Phenol, 3-[4-(4-morpholinyl)pyrido[3',2':4,5]furo[3,2-d]pyrimidin-2-yl]- | 0.224930456 | -2.152449079 | 7.37E-05 | 0.001055022 | 1.708667125 | NEG | Down |
| 2(1H)-Pyrimidinone, 5-[3-[(1S,2S,4R)-bicyclo[2.2.1]hept-2-yloxy]-4-methoxyphenyl]tetrahydro- | 0.564202212 | -0.825715773 | 0.006607992 | 0.02665018 | 1.053013052 | POS | Down |
| trans-2,3-Dimethoxycinnamic acid | 0.55042692 | -0.861377065 | 0.034449159 | 0.091592699 | 1.177469861 | POS | Down |
| 1H-Indazole-3-carboxamide, N-[(1S)-1-(aminocarbonyl)-2-methylpropyl]-1-(cyclohexylmethyl)- | 0.626480035 | -0.67465956 | 0.008660819 | 0.032898231 | 1.103819353 | POS | Down |
| 4-(1-Phenylcyclohexyl)phenol | 0.366639338 | -1.447566509 | 0.000147566 | 0.001609763 | 1.400421312 | POS | Down |
| 3-Fluoro-10H-phenothiazine | 0.301135958 | -1.731513109 | 0.00333106 | 0.01730703 | 1.301487518 | NEG | Down |
| Pyrrolidine, 2-(diphenylmethyl)-, (2S)- | 0.182710856 | -2.452365736 | 0.000361902 | 0.003166927 | 2.26759144 | POS | Down |
| 5-Heptenoic acid, 7-[(1R,2R,3S,5S)-2-[(1E,3R)-3-(2,3-dihydro-1H-inden-2-yl)-3-hydroxy-1-propen-1-yl]-3-fluoro-5-hydroxycyclopentyl]-, (5Z)- | 0.274987949 | -1.862559701 | 0.042628513 | 0.114326538 | 1.261617455 | NEG | Down |
| Gly-His-Lys | 0.26961903 | -1.891005767 | 0.001386739 | 0.008957581 | 1.276995884 | NEG | Down |
| 7-Hydroxy-4-((3Z)-5-hydroxy-3-methylpent-3-en-1-yl)-4a,8,8-trimethyl-3-methylidenedecahydronaphthalen-2-yl .beta.-D-mannopyranoside | 0.157635014 | -2.665340074 | 0.000852634 | 0.006299635 | 2.010306088 | NEG | Down |
| 6-(Dimethylamino)naphthalene-2-carboxylic acid | 0.275185551 | -1.861523374 | 0.001473096 | 0.009393975 | 1.898482474 | NEG | Down |
| Thr-Ser-Lys | 0.488993244 | -1.032113561 | 2.79E-05 | 0.000476823 | 1.641360445 | POS | Down |
| 6-(3,4-Dihydro-2(1H)-isoquinolinyl)-3-methyl[1,2,4]triazolo[3,4-a]phthalazine | 0.63005738 | -0.666444872 | 0.000688893 | 0.005032773 | 1.168623632 | POS | Down |
| 1,3,5-Triazine-2-methanol, 4-amino-6-(phenylamino)-, 2-(4-nitrobenzoate) | 0.346097936 | -1.530747758 | 0.01055932 | 0.038138833 | 1.374527819 | POS | Down |
| (3b,4b,11b,14b)-11-Ethoxy-3,4-epoxy-14-hydroxy-12-cyathen-15-al_14-xyloside | 0.265226294 | -1.914704284 | 0.003412354 | 0.016595097 | 1.8071359 | POS | Down |
| DG(15:0/18:4(6Z,9Z,12Z,15Z)/0:0) | 0.290210555 | -1.784828106 | 0.002472937 | 0.013032796 | 1.463276047 | POS | Down |
| 3-Methyl-5-pentyl-2-furannonanoic_acid | 0.396621039 | -1.334166888 | 0.002043006 | 0.011409152 | 1.095231247 | POS | Down |
| (7R*,8R*)-3-Methoxy-3',4,7,9,9'-pentahydroxy-8,4'-oxyneolignan_4-xyloside | 0.312894657 | -1.676251073 | 0.010835985 | 0.038873925 | 1.353491916 | POS | Down |
| Glutaric acid | 0.654829326 | -0.610809162 | 0.013370174 | 0.04827456 | 1.25845411 | NEG | Down |
| Harmol | 0.233259386 | -2.099992963 | 0.029308403 | 0.086757292 | 1.38031457 | NEG | Down |
| 3-Pyridinecarbonitrile, 2-amino-5-butyl-4-(2-furanyl)-6-pentyl- | 0.180619729 | -2.46897261 | 0.00054897 | 0.004295424 | 1.556596188 | POS | Down |
| Pipecolic acid | 0.331621822 | -1.59238915 | 0.000343993 | 0.003199546 | 1.304737513 | NEG | Down |
| Heptaminol | 0.312025614 | -1.68026363 | 0.000221118 | 0.002214335 | 1.442821901 | POS | Down |
| Psoralen | 0.650673251 | -0.619994849 | 0.046415457 | 0.121729851 | 1.016460513 | NEG | Down |
| N-(1,3-Diphenyl-1H-pyrazol-5-yl)benzamide | 0.450885287 | -1.149167663 | 0.006259082 | 0.025760823 | 1.422608207 | POS | Down |
| 10E,12Z-Octadecadienoic_acid | 0.65902723 | -0.601590019 | 0.000239196 | 0.002332987 | 1.44204527 | POS | Down |
| Budesonide | 0.285622444 | -1.807818746 | 0.008123827 | 0.031302921 | 1.28152116 | POS | Down |
| 17.beta.-Estradiol 3-.beta.-D-glucuronide | 0.68489889 | -0.546037073 | 0.016501708 | 0.05665827 | 1.021277997 | NEG | Down |
| Flurazepam | 0.072342926 | -3.78900424 | 0.011160103 | 0.039433748 | 1.631446347 | POS | Down |
| Cycloate | 0.340045235 | -1.55620142 | 0.001725362 | 0.010003181 | 1.336495513 | POS | Down |
| 2-Amino-4,5-difluorobenzoic acid | 0.453626742 | -1.140422403 | 0.000429735 | 0.003797838 | 2.134086539 | NEG | Down |
| 5-[1-(Phenylmethyl)-1H-indazol-3-yl]-2-furanmethanol | 0.321658799 | -1.636396941 | 0.000144284 | 0.001590674 | 2.124529485 | POS | Down |
| 2-(4-tert-Butylphenoxy)ethanamine | 0.565741958 | -0.821783922 | 0.00183196 | 0.010459298 | 1.219201592 | POS | Down |
| 4-[(3,4,5-Trimethoxybenzoyl)amino]butanoic acid | 0.534358049 | -0.904121344 | 0.003121777 | 0.016468721 | 1.271009866 | NEG | Down |
| 1-(4-Bromobenzyl)-4-(2-pyridinylmethyl)piperazine | 0.503176466 | -0.990863647 | 0.000919827 | 0.006309633 | 1.185627505 | POS | Down |
| Glyuranolide | 0.188844345 | -2.404730516 | 0.012634871 | 0.043441069 | 1.304707433 | POS | Down |
| 2-[4-(4-Fluorophenyl)piperazin-1-yl]-6-methylpyrimidin-4(3H)-one | 0.322636597 | -1.632018001 | 0.000391185 | 0.003339939 | 1.680594557 | POS | Down |
| Pentacarboxyl_porphyrinogen_III | 0.120114025 | -3.057523476 | 0.004215548 | 0.019285787 | 1.776096018 | POS | Down |
| Leupeptin | 0.214408113 | -2.221568601 | 0.001153168 | 0.007884282 | 1.667938404 | NEG | Down |
| 3-Hydroxy-10'-apo-b,y-carotenal | 0.516590183 | -0.952907867 | 0.002325675 | 0.01241981 | 1.218836367 | POS | Down |
| Adipic acid | 0.12166621 | -3.038999542 | 1.10E-05 | 0.000299647 | 1.921130185 | NEG | Down |
| 1-(3,4-Difluorophenyl)-5-oxo-3-pyrrolidinecarboxylic acid | 0.568272158 | -0.815346062 | 0.007982459 | 0.033242104 | 1.341773521 | NEG | Down |
| 2-{[2-(3,4-Dihydroxyphenyl)-2-oxoethyl]sulfanyl}-6-methyl-4(3H)-pyrimidinone | 0.458764171 | -1.124175374 | 0.002829314 | 0.014529331 | 1.376623645 | POS | Down |
| Benz[a]anthracen-1-ol, 8-methoxy-3-methyl- | 0.686620255 | -0.542415678 | 0.002894319 | 0.015613416 | 1.33880582 | NEG | Down |
| 2-Decyl-3-hydroxypentanedioic acid | 0.119264246 | -3.067766495 | 3.47E-08 | 5.61E-06 | 1.965603153 | NEG | Down |
| 1H-Indazole-1-pentanoic acid, 3-[[[1-(aminocarbonyl)-2-methylpropyl]amino]carbonyl]- | 0.348948609 | -1.518913512 | 0.002802769 | 0.015269399 | 1.371468764 | NEG | Down |
| Tetrafluorosuccinic acid | 0.04835864 | -4.370082522 | 4.83E-08 | 6.99E-06 | 2.058259299 | NEG | Down |
| Arg-Ala | 0.417457053 | -1.26030031 | 0.006065514 | 0.025129835 | 1.093701227 | POS | Down |
| Ciprofloxacin | 0.364487304 | -1.456059531 | 7.99E-07 | 4.83E-05 | 1.713612503 | NEG | Down |
| Ethyl 4-methyl-5-[(propan-2-yl)oxy]-9H-pyrido[3,4-b]indole-3-carboxylate | 0.052331357 | -4.256180524 | 9.31E-05 | 0.001245497 | 1.944774949 | NEG | Down |
| Piperanine | 0.365141302 | -1.453473231 | 2.48E-05 | 0.000437628 | 1.510137024 | POS | Down |
| .beta.-Alanine, N-[2-[(3-hexylphenyl)amino]-2-oxoethyl]- | 0.09848911 | -3.343891974 | 0.020342442 | 0.061456206 | 1.088609791 | POS | Down |
| 5-Chloro-N-[2-(dimethylamino)ethyl]-1H-indole-2-carboxamide | 0.510565325 | -0.969832534 | 0.006692419 | 0.02695198 | 1.065911824 | POS | Down |
| apo-[3-methylcrotonoyl-CoA:carbon-dioxide_ligase_(ADP-forming)] | 0.768751882 | -0.379410058 | 0.000144401 | 0.001590674 | 1.479275984 | POS | Down |
| Tacrolimus | 0.098360329 | -3.345779623 | 0.003970944 | 0.018560748 | 1.402461565 | POS | Down |
| DG(20:3(5Z,8Z,11Z)/18:3(6Z,9Z,12Z)/0:0) | 0.496006358 | -1.011569481 | 0.00528307 | 0.022691349 | 1.032358722 | POS | Down |
| Phosphonic acid, P-[[[(1R,2S)-2-aminocyclohexyl]amino]carbonyl]-, rel- | 0.637482774 | -0.649541737 | 0.003655061 | 0.018609029 | 1.545607803 | NEG | Down |
| Dimethyl_3-methoxy-4-oxo-5-(8,11,14-pentadecatrienyl)-2-hexenedioate | 0.533170689 | -0.907330625 | 0.028776157 | 0.079787898 | 1.106828718 | POS | Down |
| LysoPC(14:1(9Z)) | 0.229019401 | -2.126458273 | 3.52E-07 | 2.95E-05 | 1.794565081 | POS | Down |
| 3-Sulfopropanoic acid | 0.303280557 | -1.721275086 | 0.000164376 | 0.001874635 | 1.77826401 | NEG | Down |
| (22E,_24x)-Ergosta-4,6,8,22-tetraen-3-one | 0.437248237 | -1.193475527 | 0.002112269 | 0.011656902 | 1.224833504 | POS | Down |
| 7-[2,6-dimethyl-8-(2-methylbutanoyloxy)-1,2,6,7,8,8a-hexahydronaphthalen-1-yl]-3,5-dihydroxyheptanoic acid | 0.146043023 | -2.775534657 | 0.017719865 | 0.055801683 | 1.60141435 | POS | Down |
| Muzanzagenin | 0.564701142 | -0.824440545 | 0.02966115 | 0.081698936 | 1.080227291 | POS | Down |
| Solacauline | 0.514633639 | -0.958382334 | 0.015598772 | 0.050949941 | 1.100103182 | POS | Down |
| cis-Geranylgeranylbixin | 0.061272095 | -4.028626015 | 0.03023222 | 0.082969431 | 1.165499698 | POS | Down |
| 4-nitroquinoline 1-oxide | 0.528988324 | -0.918692216 | 0.001669088 | 0.010367205 | 1.548282476 | NEG | Down |
| 3-Oxocyclobutanecarboxylic acid | 0.724024941 | -0.4658887 | 0.013391119 | 0.04827456 | 1.113163943 | NEG | Down |
| DG(22:6(4Z,7Z,10Z,13Z,16Z,19Z)/15:0/0:0) | 0.450527047 | -1.150314374 | 0.001082696 | 0.007151615 | 1.217849121 | POS | Down |
| (3beta,17alpha,23S)-17,23-Epoxy-3,28,29-trihydroxy-27-norlanost-8-en-24-one | 0.660954013 | -0.597378198 | 0.010989545 | 0.039116657 | 1.008864631 | POS | Down |
| Lutein 5,6-epoxide | 0.222852593 | -2.165838346 | 2.39E-11 | 4.47E-08 | 2.513610857 | POS | Down |
| Glycylserylprolylmethionylphenylalanylvalinamide | 0.604362557 | -0.726513812 | 1.59E-06 | 7.81E-05 | 1.71568971 | POS | Down |
| (R)-Byakangelicin_3'-glucoside | 0.164615506 | -2.60282786 | 0.000101699 | 0.001205365 | 1.66130167 | POS | Down |
| Paynantheine | 0.458309792 | -1.125604985 | 0.000416629 | 0.003488257 | 1.349077979 | POS | Down |
| N-[(3s,5s,7s)-Adamantan-1-yl]-1-(4-fluorobenzyl)-1H-indazole-3-carboxamide | 0.317916548 | -1.653279983 | 0.000177968 | 0.001868832 | 1.664900628 | POS | Down |
| (3aS,8aR)-1,3a,8-trimethyl-1,2,3,3a,8,8a-hexahydropyrrolo[2,3-b]indol-5-yl methylcarbamate | 0.363728379 | -1.459066604 | 0.006264283 | 0.025763356 | 1.738771646 | POS | Down |
| Penitrem A | 0.711554273 | -0.490954294 | 0.000374204 | 0.003233553 | 1.194367699 | POS | Down |
| Azlocillin | 0.35501248 | -1.494058352 | 0.003056229 | 0.016185057 | 1.428845351 | NEG | Down |
| Bixin | 0.329981313 | -1.59954377 | 5.16E-05 | 0.00073691 | 1.429635393 | POS | Down |
| Annoglabasin_F | 0.114327468 | -3.128756038 | 0.002393262 | 0.012650671 | 1.548295642 | POS | Down |
| 2-Furanacetic acid, tetrahydro-5-(2-hydroxypropyl)-.alpha.-methyl-, 2-[5-(1-carboxyethyl)tetrahydro-2-furanyl]-1-methylethyl ester | 0.159993171 | -2.64391777 | 0.000410288 | 0.003673214 | 1.495855999 | NEG | Down |
| Harpagoside | 0.146470561 | -2.771317363 | 6.49E-05 | 0.0009806 | 1.757575589 | NEG | Down |
| CRUSTECDYSONE | 0.34815658 | -1.522191803 | 0.000598741 | 0.00493445 | 1.344934399 | NEG | Down |
| 5-(Trifluoromethyl)pyridine-2-carboxylic acid | 0.517433311 | -0.950555162 | 0.001043147 | 0.007304686 | 1.27567713 | NEG | Down |
| Okaramine R | 0.440575881 | -1.182537578 | 0.002510778 | 0.013195087 | 1.174445449 | POS | Down |
| Galeopsin | 0.351830228 | -1.507048657 | 0.031171394 | 0.085051429 | 1.030139193 | POS | Down |
| N6-(2,4-Dinitrophenyl)-L-lysine | 0.525153896 | -0.929187831 | 0.001921237 | 0.011529517 | 1.486707332 | NEG | Down |
| 4-[4-[(Dimethylamino)methyl]-2,5-dimethoxyphenyl]-2-methyl-2,7-naphthyridin-1-one | 0.185945536 | -2.427047979 | 4.12E-05 | 0.000641893 | 2.335290497 | POS | Down |
| 2-[4-(Hydroxycarbamoyl)phenyl]-N-(2-hydroxyethyl)-N-phenylacetamide | 0.452694482 | -1.143390373 | 0.033865001 | 0.096154913 | 1.231705725 | NEG | Down |
| papaverine | 0.453925123 | -1.139473756 | 0.016827705 | 0.053813531 | 1.461293807 | POS | Down |
| PC-M6 | 0.244082366 | -2.034560025 | 8.42E-06 | 0.000208325 | 1.580051025 | POS | Down |
| Desoxypipradrol | 0.212752742 | -2.232750372 | 0.000301509 | 0.002745347 | 1.499265229 | POS | Down |
| Loratadine | 0.307104553 | -1.703198195 | 0.009419091 | 0.035020815 | 1.4040296 | POS | Down |
| Emetine N-oxide | 0.323712887 | -1.627213296 | 0.000678483 | 0.004976132 | 1.445755992 | POS | Down |
| Pyrithiobac | 0.514722163 | -0.95813419 | 0.005233157 | 0.024316709 | 1.018148119 | NEG | Down |
| Bilobol | 0.340141331 | -1.555793775 | 4.67E-05 | 0.000692974 | 1.595159591 | POS | Down |
| .alpha.-Hexylcinnamaldehyde | 0.206171392 | -2.278083931 | 0.000374969 | 0.003233553 | 1.473298727 | POS | Down |
| Geldanamycin, 17-demethoxy-17-[[2-(dimethylamino)ethyl]amino]- | 0.286840354 | -1.80168009 | 1.93E-05 | 0.000433266 | 1.622831363 | NEG | Down |
| Methyl angolensate | 0.349539807 | -1.51647133 | 2.46E-06 | 0.000100806 | 1.739370544 | POS | Down |
| 3-Phenylpropanoic acid | 0.242382703 | -2.044641346 | 0.000135429 | 0.001632468 | 1.394907457 | NEG | Down |
| Colchiceine | 0.237069638 | -2.07661719 | 0.003484635 | 0.016847399 | 1.706804452 | POS | Down |
| Aminopicoline | 0.121960806 | -3.035510507 | 1.11E-05 | 0.0002533 | 1.993838076 | POS | Down |
| Pepstatin A | 0.068625718 | -3.865106862 | 1.95E-12 | 5.48E-09 | 2.541755002 | POS | Down |
| 1-{4-[(Diethylamino)sulfonyl]-2-nitrophenyl}-4-piperidinecarboxylic acid | 0.287102823 | -1.800360579 | 0.015649291 | 0.054445666 | 1.125684057 | NEG | Down |
| 7-((6-Deoxy-.alpha.-L-mannopyranosyl)oxy)-5-hydroxy-2-(4-hydroxyphenyl)-4-oxo-4H-chromen-3-yl 3-O-acetyl-6-deoxy-.alpha.-L-mannopyranoside | 0.552104874 | -0.856985757 | 0.006240639 | 0.027509859 | 1.385739338 | NEG | Down |
| 1,7-Bis[(Z)-but-2-en-2-yl]-3,9-dihydroxy-4,10-dimethyl-6-oxobenzo[b][1,4]benzodioxepine-2-carboxylic acid | 0.544627858 | -0.876657316 | 0.004776132 | 0.021049844 | 1.260416763 | POS | Down |
| Sch 210972 | 0.453624229 | -1.140430395 | 0.000278757 | 0.002601419 | 1.309255532 | POS | Down |
| 4-((3R,4S)-4-Hydroxy-4-(4-hydroxy-3-methoxybenzyl)-3-(hydroxymethyl)tetrahydrofuran-2-yl)-2-methoxyphenyl .beta.-D-glucopyranoside | 0.783415116 | -0.35215113 | 0.000525995 | 0.004489744 | 1.673788651 | NEG | Down |
| N-Acetyl-S-geranylgeranyl-L-cysteine | 0.592341529 | -0.755498858 | 0.005334082 | 0.022858027 | 1.091781282 | POS | Down |
| N-(3-Hydroxyphenyl)-2-(4-isobutylphenyl)propanamide | 0.486290766 | -1.040108898 | 0.037171193 | 0.09596864 | 1.355424086 | POS | Down |
| Ikarugamycin | 0.164361894 | -2.605052236 | 0.000983302 | 0.006615797 | 1.371079172 | POS | Down |
| Withaperuvin_F | 0.529840517 | -0.916369925 | 0.002283373 | 0.012322759 | 1.113510231 | POS | Down |
| Pichromene | 0.516835865 | -0.952221908 | 0.001975851 | 0.011111444 | 1.251828373 | POS | Down |
| N-(1-Amino-3,3-dimethyl-1-oxobutan-2-yl)-1-pentyl-1H-indole-3-carboxamide | 0.114644186 | -3.1247649 | 5.86E-05 | 0.000806784 | 1.814972319 | POS | Down |
| 5-(3,4'-Dihydroxy-4,4,7,8a-tetramethyl-6'-oxospiro[2,3,4a,5,6,7-hexahydro-1H-naphthalene-8,2'-3,8-dihydrofuro[2,3-e]isoindole]-7'-yl)pentanoic acid | 0.321888952 | -1.635365035 | 1.27E-06 | 7.06E-05 | 1.873117585 | POS | Down |
| Secoisolariciresinol | 0.1726654 | -2.533949081 | 0.000233268 | 0.00238785 | 1.673637119 | NEG | Down |
| 4-Hydroxyisoleucine | 0.333260602 | -1.585277321 | 0.000373887 | 0.00341405 | 1.296539249 | NEG | Down |
| N,N'-Dicarbobenzyloxy-L-ornithine | 0.404857363 | -1.304514378 | 0.012379161 | 0.042845621 | 1.18734763 | POS | Down |
| Betamethasone-17-valerate | 0.348592491 | -1.520386604 | 0.012481094 | 0.043044069 | 1.028886074 | POS | Down |
| 2-Propenoic acid, 3-[2-(.beta.-D-glucopyranosyloxy)-4-methoxyphenyl]-, (2Z)- | 0.501845332 | -0.9946853 | 5.19E-05 | 0.000841502 | 1.44827313 | NEG | Down |
| (1R,3R,4S,5R)-1,3,4-trihydroxy-5-[(E)-3-(4-hydroxyphenyl)prop-2-enoyl]oxycyclohexane-1-carboxylic acid | 0.599807708 | -0.737428032 | 0.00336445 | 0.017431083 | 1.492807477 | NEG | Down |
| VERAPAMIL | 0.409853933 | -1.286818253 | 6.96E-05 | 0.001017719 | 1.665015002 | NEG | Down |
| Myristic acid | 0.516601621 | -0.952875925 | 0.002347767 | 0.013429424 | 1.452370517 | NEG | Down |
| N-(4-Nitrophenyl)-2-phenoxyacetamide | 0.649607554 | -0.622359686 | 0.008336829 | 0.031862698 | 1.058265269 | POS | Down |
| Methylsuccinic acid | 0.637935686 | -0.648517109 | 0.010988951 | 0.042015276 | 1.293232156 | NEG | Down |
| Muricarpone B | 0.528662897 | -0.919580018 | 0.013565885 | 0.048771529 | 1.03083528 | NEG | Down |
| Acutoside_A | 0.276571975 | -1.854273118 | 0.007356113 | 0.029041915 | 1.229438867 | POS | Down |
| Benthiavalicarb-isopropyl | 0.565501621 | -0.822396936 | 0.001586157 | 0.009511812 | 1.172699621 | POS | Down |
| 16-Phenyltetranorprostaglandin E2 | 0.546409382 | -0.87194584 | 0.02300137 | 0.0725095 | 1.144560454 | NEG | Down |
| 2-(4-Hydroxyphenyl)ethyl 6-O-((2-oxo-2,3-dihydro-1H-indol-3-yl)acetyl)-.beta.-D-glucopyranoside | 0.428739768 | -1.221825855 | 0.000714774 | 0.005565316 | 1.734556607 | NEG | Down |
| Epiafzelechin (2R,3R)(-) | 0.431383754 | -1.212956248 | 0.001700396 | 0.010514148 | 1.207657344 | NEG | Down |
| (2E,6E,11E,13E)-18-(2,6-Dioxopiperidin-4-yl)-9-hydroxy-8-methoxy-10,12,14-trimethyl-15-oxooctadeca-2,6,11,13-tetraenoic acid | 0.259989827 | -1.943472922 | 1.07E-05 | 0.000247101 | 1.52198713 | POS | Down |
| N-Cyclooctyl-2-phenoxypropanamide | 0.33471118 | -1.579011353 | 0.049461848 | 0.117694478 | 1.052242422 | POS | Down |
| Antibiotic TAN 1446A | 0.387270963 | -1.36858476 | 0.005973487 | 0.026696143 | 1.450712677 | NEG | Down |
| Mibefradil | 0.410981026 | -1.282856304 | 0.000229308 | 0.002264064 | 1.668525586 | POS | Down |
| Benzylfentanyl | 0.46251918 | -1.112414901 | 9.04E-05 | 0.001096471 | 1.44854355 | POS | Down |
| Piperidolate | 0.550567985 | -0.861007374 | 0.001667111 | 0.009812619 | 1.141599387 | POS | Down |
| 2,6-Dimethylpyrazine | 0.121392408 | -3.042249902 | 9.83E-06 | 0.000232487 | 2.001397765 | POS | Down |
| 2,5-Dimethylpyrazine | 0.120411859 | -3.053950607 | 9.49E-06 | 0.000227816 | 2.005908277 | POS | Down |
| 2-{[((2Z)-4-Phenyl-1,3-thiazol-2(3H)-ylidene)amino]carbonyl}benzoic acid | 0.679395286 | -0.557676887 | 0.000756948 | 0.005771071 | 1.26702809 | NEG | Down |
| 2-Aminomuconate | 0.446403703 | -1.163579101 | 0.01286831 | 0.047252573 | 1.994607372 | NEG | Down |
| (3S,5S)-Carbapenam-3-carboxylic acid | 0.238444887 | -2.068272249 | 0.001170202 | 0.007591451 | 1.396843221 | POS | Down |
| Isoniazid pyruvate | 0.612577206 | -0.707036411 | 0.019874032 | 0.065127765 | 1.338077524 | NEG | Down |
| gamma-Glutamyl-gamma-aminobutyraldehyde | 0.448963898 | -1.155328655 | 0.000819024 | 0.005773249 | 1.688186097 | POS | Down |
| Dethiobiotin | 0.43974701 | -1.185254327 | 0.000522856 | 0.004469891 | 1.703706418 | NEG | Down |
| Crepenynate | 0.232364297 | -2.10553968 | 0.035500915 | 0.099667216 | 1.325476621 | NEG | Down |
| Dihydrofolate | 0.23362305 | -2.097745474 | 0.002045336 | 0.012024828 | 1.591658405 | NEG | Down |
| Orotic acid | 0.206016633 | -2.279167275 | 3.61E-08 | 5.61E-06 | 2.144290676 | NEG | Down |
| 3,7,4'-Tri-O-methylquercetin | 0.259988293 | -1.943481435 | 0.030061314 | 0.088367402 | 1.208911579 | NEG | Down |
| Peganine | 0.337584049 | -1.566681358 | 0.046188233 | 0.121539836 | 1.360007898 | NEG | Down |
| 1-Myristoyl-sn-glycero-3-phosphocholine (LPC(14:0/0:0)) | 0.171060336 | -2.547422812 | 1.14E-05 | 0.00025823 | 1.531415413 | POS | Down |
| Deoxycytidine | 0.517787026 | -0.949569279 | 0.000548772 | 0.004295424 | 1.455745295 | POS | Down |
| Oxypurinol | 0.593779349 | -0.752001177 | 0.001661902 | 0.009807318 | 1.048409944 | POS | Down |
| 2-Ethylpyrazine | 0.121392408 | -3.042249902 | 9.83E-06 | 0.000232487 | 2.001397765 | POS | Down |
| Indoleacetic acid | 0.488954863 | -1.032226805 | 0.000763604 | 0.005805712 | 1.222405074 | NEG | Down |
| Sphingosine | 0.26403683 | -1.921188913 | 0.044856246 | 0.109427181 | 1.57336481 | POS | Down |
| Xanthine | 0.469939342 | -1.089453544 | 0.001010141 | 0.006739869 | 1.142160245 | POS | Down |
| L-鈥婰eucyl-鈥婰-鈥媋lanine | 0.290893276 | -1.781438148 | 0.039716254 | 0.100170765 | 1.429137967 | POS | Down |
| 5-Hydroxyindole-3-acetic acid | 0.462594974 | -1.112178504 | 0.006509009 | 0.028532713 | 1.156143238 | NEG | Down |
| Hypoxanthine | 0.725985287 | -0.461987785 | 0.00800337 | 0.030885933 | 1.023469232 | POS | Down |
| 1-Methylxanthine | 0.406003443 | -1.300436134 | 0.002723332 | 0.014940274 | 1.152519374 | NEG | Down |
| Allolithocholic acid | 0.513136644 | -0.96258504 | 0.000218861 | 0.002300745 | 1.306505468 | NEG | Down |
| 2-Methylglutaric acid | 0.12166621 | -3.038999542 | 1.10E-05 | 0.000299647 | 1.921130185 | NEG | Down |
| 3,3-Dimethylglutaric acid | 0.428800243 | -1.22162237 | 0.001014157 | 0.007185731 | 1.211922085 | NEG | Down |
| Hygric acid | 0.539177103 | -0.891168863 | 0.00051576 | 0.004121679 | 1.373263489 | POS | Down |
| Cholest-4-en-3-one | 0.153157963 | -2.706907722 | 7.05E-06 | 0.000188876 | 1.937894711 | POS | Down |
| Methyl beta-D-glucopyranoside | 0.393904935 | -1.344080604 | 0.000232473 | 0.00238785 | 1.744312816 | NEG | Down |
| Glu-Arg | 0.57463509 | -0.799282001 | 0.001978257 | 0.011113847 | 1.256103822 | POS | Down |
| 3-Amino-4-hydroxybenzoic acid | 0.450526835 | -1.150315053 | 0.001550213 | 0.0093848 | 1.495748777 | POS | Down |
| (-)-Camphoric acid | 0.541126653 | -0.885961792 | 0.001972216 | 0.011682405 | 1.323539935 | NEG | Down |
| Phe-Val | 0.39574018 | -1.337374542 | 0.040910734 | 0.102331479 | 1.250369721 | POS | Down |
| 2'-Deoxyuridine | 0.476268111 | -1.070154141 | 0.037291956 | 0.103584579 | 1.126328341 | NEG | Down |
| 3-Methylglutaric acid | 0.121603964 | -3.039737837 | 1.11E-05 | 0.000299647 | 1.919349423 | NEG | Down |
| Thymine | 0.585095705 | -0.773255468 | 0.001996055 | 0.011798185 | 1.180170451 | NEG | Down |
| Isolithocholic acid | 0.51201161 | -0.965751572 | 0.000215666 | 0.002293072 | 1.307730199 | NEG | Down |
| 2-Pyrimidinylacetic acid | 0.580836022 | -0.783797168 | 0.015947868 | 0.05520493 | 1.192937507 | NEG | Down |
| 1,3-Dimethyluric acid | 0.617880198 | -0.694600956 | 9.15E-07 | 5.11E-05 | 2.096250243 | NEG | Down |
| 4'-Hydroxy-3'-methylacetophenone | 0.242382703 | -2.044641346 | 0.000135429 | 0.001632468 | 1.394907457 | NEG | Down |
| 4鈥?Hydroxy-2鈥?methylacetophenone | 0.242157922 | -2.045979893 | 0.000136014 | 0.001632468 | 1.394421885 | NEG | Down |
| 3-Methylxanthine | 0.406003411 | -1.300436248 | 0.002722606 | 0.014940274 | 1.152335947 | NEG | Down |
| Lithocholic acid | 0.51201161 | -0.965751572 | 0.000215666 | 0.002293072 | 1.307730199 | NEG | Down |
| LPC(13:0) | 0.405648727 | -1.301697136 | 5.00E-05 | 0.00081994 | 1.747041228 | NEG | Down |
| 4-Allylcatechol | 0.242382703 | -2.044641346 | 0.000135429 | 0.001632468 | 1.394907457 | NEG | Down |
| 2'-Hydroxy-4'-methylacetophenone | 0.242157922 | -2.045979893 | 0.000136014 | 0.001632468 | 1.394421885 | NEG | Down |
| Pro-Phe | 0.630608767 | -0.665182868 | 0.001785989 | 0.010280414 | 1.268744086 | POS | Down |
| 2'-Hydroxy-5'-methylacetophenone | 0.242382703 | -2.044641346 | 0.000135429 | 0.001632468 | 1.394907457 | NEG | Down |
| 7-Methylxanthine | 0.406000603 | -1.300446223 | 0.002722425 | 0.014940274 | 1.152308971 | NEG | Down |
| Sph(d18:0) | 0.268551837 | -1.896727504 | 0.019277987 | 0.059053287 | 1.628915964 | POS | Down |
| 2,6-Dimethyl-4-hydroxybenzaldehyde | 0.242153385 | -2.046006923 | 0.000133826 | 0.001632468 | 1.395769404 | NEG | Down |
| N-Acetylmuramic acid | 0.451595347 | -1.146897473 | 1.93E-06 | 8.77E-05 | 1.780567658 | NEG | Down |
| gamma-Glutamylleucine | 0.586165489 | -0.770620064 | 0.038891141 | 0.098767708 | 1.103274112 | POS | Down |
| 1-Methylguanine | 0.580626871 | -0.784316755 | 0.032477346 | 0.087551694 | 1.259519726 | POS | Down |
| 3-Methyluridine | 0.668244455 | -0.581552133 | 0.005138896 | 0.024041284 | 1.191315122 | NEG | Down |
| 4-Oxohexanoic acid | 0.402275718 | -1.313743437 | 0.00286877 | 0.015536577 | 1.130479612 | NEG | Down |
| Phenazine-1-carboxylic acid | 0.290207629 | -1.784842647 | 3.41E-05 | 0.000621029 | 1.443121931 | NEG | Down |
| Suberic acid | 0.357343939 | -1.484614775 | 0.001372764 | 0.008909187 | 1.202501788 | NEG | Down |
| 3-Pyridol | 0.298956739 | -1.741991362 | 0.004041217 | 0.020180435 | 1.412898438 | NEG | Down |
| Allopurinol | 0.727878012 | -0.458231411 | 0.008245209 | 0.031640425 | 1.019390147 | POS | Down |
| 1-Methyl-6-oxo-1,6-dihydropyridine-3-carboxylic acid | 0.450662895 | -1.149879423 | 0.001578025 | 0.009491806 | 1.494516159 | POS | Down |
| Cytisine | 0.202377558 | -2.304878781 | 0.015924593 | 0.051713505 | 1.416331629 | POS | Down |
| Azelaic acid | 0.452030935 | -1.145506587 | 0.025918766 | 0.079241078 | 1.200951978 | NEG | Down |
| Bisindolylmaleimide VIII (acetate) | 0.329416912 | -1.60201347 | 0.013903872 | 0.049597394 | 1.246527201 | NEG | Down |
| Nicotinamide riboside (NR) | 0.350460762 | -1.512675169 | 0.037539713 | 0.096520872 | 1.446073399 | POS | Down |
| Prostaglandin F2alpha (PGF2a) | 0.466281302 | -1.100727516 | 0.000267073 | 0.002640464 | 1.445952872 | NEG | Down |
| Acetoin | 0.710855652 | -0.492371461 | 0.007428949 | 0.03136011 | 1.108620989 | NEG | Down |
| 1,7-Dimethyluric acid | 0.617878688 | -0.694604483 | 8.80E-07 | 5.04E-05 | 2.096128556 | NEG | Down |
| 1-Methylpseudouridine | 0.668244455 | -0.581552133 | 0.005138896 | 0.024041284 | 1.191315122 | NEG | Down |
| LPE(14:0) | 0.597060296 | -0.744051462 | 0.014869152 | 0.049109286 | 1.060302381 | POS | Down |
| 3-(5-methoxy-1H-indol-3-yl)propanoic acid | 0.238535771 | -2.067722463 | 0.004108977 | 0.020403835 | 1.32131696 | NEG | Down |
| Fluorofenidone | 0.270895416 | -1.884192114 | 5.72E-05 | 0.000796816 | 1.599721693 | POS | Down |
| Cinnamylideneacetic acid | 0.033722044 | -4.890164197 | 1.11E-07 | 1.20E-05 | 2.019499805 | NEG | Down |
| Hypaphorine | 0.723955472 | -0.46602713 | 0.009274268 | 0.034591553 | 1.192408908 | POS | Down |
| 2-Oxoadipic acid | 0.669430565 | -0.578993673 | 0.035573418 | 0.099667216 | 1.097794422 | NEG | Down |
| 4-(4-Methyl-1-piperazinyl)butanoic acid | 0.492047191 | -1.023131408 | 0.000987774 | 0.006630006 | 1.52046599 | POS | Down |
| Isonicotinamide | 0.369201724 | -1.437518806 | 0.002954234 | 0.015060695 | 1.647021471 | POS | Down |
| Leu-Gly | 0.613699026 | -0.7043968 | 0.001943153 | 0.011015777 | 1.309411382 | POS | Down |
| Methyl beta-D-Galactopyranoside | 0.393904935 | -1.344080604 | 0.000232473 | 0.00238785 | 1.744312816 | NEG | Down |
| N-Benzylacetamidine | 0.334833316 | -1.578485011 | 3.97E-06 | 0.00012821 | 1.684049949 | POS | Down |
| Glycerophospho-N-palmitoylethanolamine | 0.36051026 | -1.471887776 | 1.62E-06 | 7.91E-05 | 1.955400041 | POS | Down |
| 4-Nitrophenol | 0.353273008 | -1.501144571 | 0.012610965 | 0.046519165 | 1.411500232 | NEG | Down |
| Neostigmine cation | 0.494399529 | -1.016250726 | 0.004427439 | 0.020026853 | 1.525690686 | POS | Down |
| 5,6-Dihydrouridine | 0.35589258 | -1.49048624 | 0.000677385 | 0.005357288 | 1.263186532 | NEG | Down |
| Pentylenetetrazole | 0.504650829 | -0.98664257 | 0.02808535 | 0.07842122 | 1.237333775 | POS | Down |
| Ala-Val | 0.65663781 | -0.60683027 | 0.009825259 | 0.038744223 | 1.145050952 | NEG | Down |
| 2-(2,6-Dimethylmorpholin-4-yl)ethanol | 0.494537437 | -1.015848356 | 0.000458633 | 0.00376326 | 1.285425168 | POS | Down |
| 3-Hydroxyacetaminophen | 0.602872501 | -0.73007517 | 0.018996861 | 0.058415087 | 1.076836551 | POS | Down |
| Pro-Ile | 0.42456931 | -1.235928005 | 6.80E-05 | 0.001013718 | 1.708833993 | NEG | Down |
| Ile-Pro | 0.473255684 | -1.079308261 | 7.76E-05 | 0.0009842 | 1.468911304 | POS | Down |
| 3-hydroxy-C4-homoserine lactone | 0.541191639 | -0.885788543 | 0.016700211 | 0.05709021 | 1.327122298 | NEG | Down |
| 2-Cyanoamino-4,6-dihydroxypyrimidine | 0.670697345 | -0.576266203 | 0.002542809 | 0.014161928 | 1.125096474 | NEG | Down |
| 1,1-Dimethyl-4-phenylpiperazin-1-ium cation | 0.238358218 | -2.068796729 | 0.000598732 | 0.004541124 | 1.797712544 | POS | Down |
| Methyl acetoacetate | 0.586155319 | -0.770645095 | 0.005300618 | 0.022749327 | 1.021393369 | POS | Down |
| 5-(2-Furyl)-4H-1,2,4-triazol-3-amine | 0.677983877 | -0.560677129 | 0.002835505 | 0.014547824 | 1.121708561 | POS | Down |
| Cordycepin | 0.296401433 | -1.75437567 | 2.98E-06 | 0.000110733 | 1.742701104 | POS | Down |
| 4,6-Dihydroxypyrimidine | 0.706074809 | -0.50210705 | 0.004417813 | 0.02135859 | 1.044727028 | NEG | Down |
| 11-dehydro_TXB2 | 0.219322813 | -2.188872215 | 3.19E-05 | 0.000593799 | 1.890654256 | NEG | Down |
| LPE(P-16:0) | 0.220955017 | -2.178175406 | 0.001869926 | 0.010643611 | 2.042423875 | POS | Down |
| Methanone, [1-(5-fluoropentyl)-7-hydroxy-1H-indol-3-yl]-1-naphthalenyl- | 0.172938962 | -2.531665161 | 0.000473918 | 0.003836418 | 1.659825819 | POS | Down |
| Forskolin | 0.442270544 | -1.176998934 | 0.003653439 | 0.018609029 | 1.184313224 | NEG | Down |
| 1-(2,5-Dimethylphenoxy)-3-(4-morpholinyl)-2-propanol | 0.385956411 | -1.373490173 | 0.000194038 | 0.00199653 | 1.777747415 | POS | Down |
| Thr-Pro | 0.667471454 | -0.583221956 | 0.004693741 | 0.020847599 | 1.145409429 | POS | Down |
| Nitracaine | 0.226815316 | -2.14041003 | 0.001653146 | 0.009777772 | 1.23886845 | POS | Down |
| Ala-Pro | 0.58311342 | -0.778151569 | 0.000222678 | 0.00222203 | 1.439843141 | POS | Down |
| Ala-Thr | 0.312907447 | -1.676192101 | 0.022522076 | 0.066454319 | 1.608634721 | POS | Down |
| 4-(Trifluoromethyl)piperidine | 0.178430228 | -2.486568051 | 6.20E-07 | 4.24E-05 | 1.843832169 | POS | Down |
| Val-Pro | 0.397470039 | -1.331081978 | 1.09E-05 | 0.000250027 | 1.716305292 | POS | Down |
| 24-epi-brassinolide | 0.210578792 | -2.247567951 | 0.041165725 | 0.11158185 | 1.408213127 | NEG | Down |
| Picolinic acid | 0.548228889 | -0.867149741 | 0.007753267 | 0.032484534 | 1.149677562 | NEG | Down |
| Flavin adenine dinucleotide (FAD) | 0.336847094 | -1.569834243 | 1.42E-05 | 0.00035257 | 1.560721462 | NEG | Down |
| N-Ethyl-N-methylcathinone | 0.577477579 | -0.792163161 | 0.008174423 | 0.031433202 | 1.132847413 | POS | Down |
| CROTONIC ACID | 0.579921622 | -0.786070166 | 0.033822691 | 0.096084411 | 1.360315512 | NEG | Down |
| 2-Hydroxy-4,6-dimethylpyrimidine | 0.330941561 | -1.595351611 | 0.000113985 | 0.001334097 | 1.735036533 | POS | Down |
| 7alpha-Hydroxy-5beta-cholan-24-oic acid | 0.434773076 | -1.201665494 | 8.00E-05 | 0.001120973 | 1.364599452 | NEG | Down |
| 2-Oxiraneoctanoic acid, .eta.-hydroxy-3-octyl- | 0.813385115 | -0.297989506 | 0.014359272 | 0.050826089 | 1.226582711 | NEG | Down |
| 4-Hydroxy-2',4',6'-trimethoxychalcone | 0.5328049 | -0.908320744 | 3.55E-05 | 0.000638999 | 1.350777712 | NEG | Down |
| (2S,3aS,7aS)-Octahydro-1H-indole-2-carboxylic acid | 0.803856777 | -0.314989616 | 0.020177159 | 0.061017518 | 1.001188228 | POS | Down |
| Androsterone sulfate | 0.77734474 | -0.363373541 | 0.020075833 | 0.06549368 | 1.078528327 | NEG | Down |
| Pimonidazole | 0.308339357 | -1.697409044 | 0.000586152 | 0.004486379 | 2.19144293 | POS | Down |
| Lys-Ala | 0.458712813 | -1.124336889 | 0.024218672 | 0.070315503 | 1.274489342 | POS | Down |
| Pro-Lys | 0.435316275 | -1.199864137 | 0.00020918 | 0.002254628 | 1.815888597 | NEG | Down |
| TRICARBALLYLIC ACID | 0.283495969 | -1.818599874 | 2.36E-05 | 0.000487616 | 2.050992163 | NEG | Down |
| Nifedipine | 0.698831591 | -0.516983267 | 0.000417531 | 0.003719884 | 1.365155614 | NEG | Down |
| 20-hydroxy-PGE2 | 0.295784988 | -1.757379261 | 0.000602441 | 0.0049501 | 1.359131676 | NEG | Down |
| 1.alpha.-Methyl-5.alpha.-androstan-3.alpha.,17.beta.-diol glucuronide | 0.215662408 | -2.213153371 | 0.000930816 | 0.006749808 | 1.767067308 | NEG | Down |
| Gln-Val | 0.425400741 | -1.233105549 | 0.002155876 | 0.011816305 | 1.610046377 | POS | Down |
| Glycylleucine | 0.562103809 | -0.831091504 | 0.014184833 | 0.050305822 | 1.473030189 | NEG | Down |
| 3,4-Dihydro-3-oxo-2H-(1,4)-benzoxazin-2-ylacetic acid | 0.343029134 | -1.543596982 | 0.001817646 | 0.011029165 | 1.456885055 | NEG | Down |
| Met-Pro | 0.376543168 | -1.409112825 | 0.002294996 | 0.012338075 | 1.230329997 | POS | Down |
| 2-((2R)-2-Hydroxycyclohexyl)acetic acid | 0.582250241 | -0.780288762 | 0.000744834 | 0.005710397 | 1.158389835 | NEG | Down |
| Leucine | 0.501653136 | -0.995237926 | 0.000280774 | 0.002613575 | 1.698150241 | POS | Down |
| Aspergamide B | 0.396273793 | -1.335430537 | 5.50E-07 | 4.01E-05 | 1.933629338 | POS | Down |
| Rotundine | 0.157896041 | -2.662953096 | 2.04E-05 | 0.000389224 | 1.629542436 | POS | Down |
| Norleucine | 0.515935769 | -0.954736624 | 0.000426356 | 0.003548549 | 1.651100387 | POS | Down |
| [6]-Gingerdiol_3,5-diacetate | 0.127298728 | -2.973710088 | 2.52E-05 | 0.000503788 | 1.721696962 | NEG | Down |
| D-erythro-Sphingosine C-20 | 0.300866075 | -1.732806652 | 2.45E-05 | 0.000432618 | 1.644534363 | POS | Down |
| 6-Methoxy-3-pyridinamine | 0.315352409 | -1.66496314 | 0.000173941 | 0.001842104 | 1.946088063 | POS | Down |
| 2-(Methylamino)-1-(morpholin-4-yl)ethan-1-one | 0.132516206 | -2.91575929 | 8.01E-05 | 0.001006088 | 1.606665617 | POS | Down |
| Simvastatin | 0.243656197 | -2.037081177 | 0.000333435 | 0.00296868 | 1.389624306 | POS | Down |
| Codeine | 0.215973435 | -2.211074223 | 6.62E-05 | 0.000870077 | 1.535125464 | POS | Down |
| Secobarbital | 0.69180821 | -0.53155596 | 0.014280685 | 0.047670167 | 1.114110685 | POS | Down |
| Xanthinol | 0.132792612 | -2.912753212 | 2.48E-06 | 0.000100806 | 1.818035974 | POS | Down |
| Glu-Val | 0.44542812 | -1.166735456 | 0.015694091 | 0.054532502 | 1.586025723 | NEG | Down |
| 3-Oxo-1,8-octanedicarboxylic acid | 0.376588459 | -1.408939309 | 0.007314293 | 0.030975864 | 1.433986483 | NEG | Down |
| 20-carboxy-LTB4 | 0.377973964 | -1.403641233 | 0.009751564 | 0.038564278 | 1.120157242 | NEG | Down |
| 6-Amino-1-(2-methoxyethyl)-2,4(1H,3H)-pyrimidinedione | 0.385816112 | -1.374014701 | 5.17E-05 | 0.00073691 | 1.8405673 | POS | Down |
| Gln-Ile | 0.577664818 | -0.791695463 | 0.02950083 | 0.081442585 | 1.123064408 | POS | Down |
| 9H-Purine-2,6-diamine, N6-(1R,2S,4S)-bicyclo[2.2.1]hept-2-yl-N2-phenyl-, rel- | 0.448580617 | -1.156560813 | 0.000277882 | 0.002599035 | 1.359436001 | POS | Down |
| 2-oxindole-3-acetate | 0.461567057 | -1.115387834 | 0.006337195 | 0.027913109 | 1.158694848 | NEG | Down |
| 2-(Hydroxymethyl)-5-methyl-4H,7H-[1,2,4]triazolo[1,5-a]pyrimidin-7-one | 0.214446618 | -2.221309533 | 0.003933996 | 0.019749019 | 1.086519839 | NEG | Down |
| 23S,25-dihydroxyvitamin_D3 | 0.688193429 | -0.539113979 | 0.014028721 | 0.047137174 | 1.026884537 | POS | Down |
| Methanone, [1-(5-fluoropentyl)-6-nitro-1H-indol-3-yl]-1-naphthalenyl- | 0.36981295 | -1.435132351 | 0.003239435 | 0.016007187 | 1.245329552 | POS | Down |
| 4-Oxododecanedioic acid | 0.158557663 | -2.656920491 | 2.74E-06 | 0.000113058 | 1.835746954 | NEG | Down |
| N,N'-Diacetylcystine | 0.553322855 | -0.85380658 | 3.58E-05 | 0.000640795 | 1.436855089 | NEG | Down |
| Isoproturon | 0.723427379 | -0.467079896 | 0.004811771 | 0.021152216 | 1.248016072 | POS | Down |
| 4,5-Diaminopentanoic acid | 0.610292002 | -0.712428412 | 0.000987412 | 0.006630006 | 1.268219778 | POS | Down |
| 1-[(Dimethylamino)carbonyl]-3-piperidinecarboxylic acid | 0.972520552 | -0.040199357 | 0.019942221 | 0.060461628 | 1.097326346 | POS | Down |
| Cafestol | 0.681295877 | -0.553646619 | 0.002160528 | 0.011830261 | 1.200216855 | POS | Down |
| Ser-Val | 0.571491234 | -0.807196725 | 0.042423853 | 0.105133307 | 1.141383012 | POS | Down |
| Paromomycin | 0.278817072 | -1.842609196 | 7.23E-06 | 0.000188876 | 1.643415785 | POS | Down |
| 2-[4-[(2-Phenyl-1H-imidazol-5-yl)methyl]piperazino]pyrimidine | 0.478176779 | -1.064384022 | 9.53E-05 | 0.001265977 | 1.395967952 | NEG | Down |
| Methyl 1-piperazinecarboxylate | 0.110582835 | -3.176800637 | 1.68E-05 | 0.000339155 | 1.98230866 | POS | Down |
| 7-Methylguanine | 0.579963246 | -0.785966621 | 0.032462385 | 0.087551694 | 1.262131144 | POS | Down |
| [(1S)-1-Benzyl-2-[[5-(3-methyl-2H-indazol-5-yl)-3-pyridyl]oxy]ethyl]amine | 0.076209754 | -3.713880531 | 7.77E-07 | 4.80E-05 | 1.863270451 | NEG | Down |
| 9Z,11E,13E-Octadecatrienoic acid methyl ester | 0.301532447 | -1.729614842 | 0.002010781 | 0.011246795 | 1.826679598 | POS | Down |
| Methanone, [1-(3-fluoropentyl)-1H-indol-3-yl]-1-naphthalenyl- | 0.460838114 | -1.117668054 | 0.000108202 | 0.00127171 | 1.513001552 | POS | Down |
| Arg-Pro | 0.557451723 | -0.843081226 | 0.001789043 | 0.010287456 | 1.300853969 | POS | Down |
| 6-Methyl-3-phenylpyrimido[5,4-e][1,2,4]triazine-5,7(6H,8H)-dione | 0.694191334 | -0.526594739 | 0.045755203 | 0.120795789 | 1.015337721 | NEG | Down |
| Glycine, 1,1'-(1,8-dioxo-1,8-octanediyl)bis[glycyl- | 0.278322497 | -1.845170565 | 0.001883299 | 0.011376368 | 1.478941054 | NEG | Down |
| Deoxyinosine | 0.526195095 | -0.926330295 | 0.025581453 | 0.07295259 | 1.281634929 | POS | Down |
| Ethenodeoxyadenosine | 0.634110163 | -0.657194596 | 0.012269115 | 0.042574358 | 1.293728299 | POS | Down |
| N-Demethylsambutoxin | 0.408233325 | -1.292534136 | 0.000952659 | 0.006845447 | 1.319104187 | NEG | Down |
| N-Methyl-L-threonine | 0.480412169 | -1.0576554 | 0.000899858 | 0.00620295 | 1.301451646 | POS | Down |
| Lys-Pro | 0.48605468 | -1.040809471 | 2.60E-05 | 0.000451761 | 1.540932781 | POS | Down |
| Butanoic acid | 0.700468377 | -0.513608175 | 0.005964163 | 0.026676164 | 1.13066025 | NEG | Down |
| 5-Isopropyl-5-methylhydantoin | 0.267313733 | -1.903394139 | 0.000237773 | 0.002323149 | 1.444821973 | POS | Down |
| LPE(O-18:2) | 0.347268936 | -1.52587473 | 1.22E-07 | 1.37E-05 | 1.960169247 | POS | Down |
| Glu-Lys | 0.688208322 | -0.539082757 | 0.005949813 | 0.024833617 | 1.033240301 | POS | Down |
| (Methylsulfanyl)heptyl glucosinolate | 0.282935718 | -1.821453778 | 0.00918925 | 0.037033217 | 1.50831291 | NEG | Down |
| Hexacyclinic acid | 0.183962278 | -2.442518127 | 2.84E-05 | 0.000546844 | 2.05989989 | NEG | Down |
| [1,2,4]Triazolo[4,3-a]quinazolin-5(1H)-one, 2,4-dihydro-4-methyl-1-thioxo- | 0.752161637 | -0.410885369 | 0.010201289 | 0.037190683 | 1.01111214 | POS | Down |
| 2-(1,3-Thiazol-4-yl)-1H-benzimidazol-5-amine | 0.288873239 | -1.791491537 | 0.023546858 | 0.073543795 | 1.317389202 | NEG | Down |
| 12-Methyltridecanoic acid | 0.516601621 | -0.952875925 | 0.002347767 | 0.013429424 | 1.452370517 | NEG | Down |
| Di(3,7-dimethyl-1-octyl) phthalate | 0.434526895 | -1.202482621 | 0.000411708 | 0.003467733 | 1.25448465 | POS | Down |
| (3S,3'R,5R,6R)-7',8'-Didehydro-3,6-epoxy-5,6-dihydro-beta,beta-carotene-3',5-diol | 0.271538957 | -1.8807689 | 1.19E-07 | 1.36E-05 | 2.227867073 | POS | Down |
| Octadienedioylcarnitine | 0.682445849 | -0.55121352 | 0.003228558 | 0.015994741 | 1.331638547 | POS | Down |
| 2,2-Dimethyl-N-[3-(trifluoromethyl)phenyl]propanamide | 0.215501581 | -2.214229644 | 5.85E-05 | 0.000806784 | 2.134217901 | POS | Down |
| 4-(2-Trifluoromethylphenyl)piperidine | 0.568471879 | -0.81483911 | 0.005602631 | 0.023701492 | 1.223441291 | POS | Down |
| Heptane-1-thiol | 0.490630051 | -1.027292493 | 0.000300844 | 0.002743738 | 1.653162204 | POS | Down |
| HDMBOA (not validated, isomer of 871) | 0.606861983 | -0.72055965 | 6.94E-05 | 0.000900664 | 1.586319112 | POS | Down |
| 3-(Trifluoromethyl)phenylalanine | 2.240247012 | 1.163657814 | 0.012748864 | 0.046907969 | 1.613932275 | NEG | Up |
| Benzenesulfonic acid | 17.66227423 | 4.142599214 | 0.000205613 | 0.002238127 | 1.204583362 | NEG | Up |
| Alsterpaullone | 57.47854789 | 5.84495171 | 0.017530213 | 0.055450825 | 1.374826124 | POS | Up |
| 7-Chloro-5-(1H-pyrrol-2-yl)-1,3-dihydro-1,4-benzodiazepin-2-one | 204.5077448 | 7.676011669 | 0.02479232 | 0.076649822 | 1.167895079 | NEG | Up |
| Lotaustralin | 17.10378799 | 4.096243971 | 0.003818689 | 0.018119421 | 1.040172237 | POS | Up |
| 3-[(Ethylanilino)methyl]benzenesulfonic acid | 6.207240098 | 2.633951951 | 0.00055295 | 0.004654773 | 1.713535984 | NEG | Up |
| trans-Hexadec-2-enoyl_carnitine | 14.18800329 | 3.826599665 | 0.033673597 | 0.089956381 | 1.304332534 | POS | Up |
| 3-[4-(4-Hydroxy-2-quinazolinyl)benzyl]-2,4-imidazolidinedione | 15.09405553 | 3.915908582 | 0.000152615 | 0.001769882 | 1.435779586 | NEG | Up |
| Tetraethylene glycol monomethyl ether | 1.686336373 | 0.753892339 | 0.033220691 | 0.088915598 | 1.113969101 | POS | Up |
| Thieno[2,3-b]pyridine-5-carbonitrile, 6,7-dihydro-4-hydroxy-3-(2'-hydroxy[1,1'-biphenyl]-4-yl)-6-oxo- | 10.91613442 | 3.44839016 | 0.017932789 | 0.059997893 | 1.266593813 | NEG | Up |
| 2-Methyl-N-(4-methylphenyl)benzenesulfonamide | 3.207752349 | 1.681562764 | 0.016904961 | 0.057420926 | 1.189281276 | NEG | Up |
| Ethyl (2Z)-2-cyano-3-(1H-indol-3-yl)prop-2-enoate | 3.497283045 | 1.806234562 | 0.031640794 | 0.091928883 | 1.073187813 | NEG | Up |
| PC(40:1) | 11.8928342 | 3.572020662 | 0.006805152 | 0.027347172 | 1.544546998 | POS | Up |
| S-Allyl-L-Cysteine | 32.63421354 | 5.028313367 | 0.031833586 | 0.086354943 | 1.254916328 | POS | Up |
| 3-Cyano-4,7-dimethylcoumarin | 3.441232291 | 1.78292528 | 0.000223433 | 0.002225611 | 1.713405926 | POS | Up |
| 4-(Methylnitrosamino)-1-(3-pyridyl-N-oxide)-1-butanol | 7.386809121 | 2.884951298 | 0.004480927 | 0.02020373 | 1.23311559 | POS | Up |
| Ursocholic acid | 26.62570903 | 4.734748039 | 4.71E-06 | 0.000168253 | 1.604164704 | NEG | Up |
| 5-Fluoropentyl-3-pyridinoylindole | 1.004303319 | 0.006195057 | 0.003400979 | 0.016571293 | 1.072477963 | POS | Up |
| 2-Amino-1-(4-methylphenyl)-4-phenyl-1H-pyrrole-3-carbonitrile | 4.217073657 | 2.07624222 | 0.031443561 | 0.085503353 | 1.037631749 | POS | Up |
| Lactate | 6.070139936 | 2.601729776 | 0.006066396 | 0.026981532 | 1.173785093 | NEG | Up |
| Benzamide, N-1H-pyrrolo[2,3-c]pyridin-5-yl- | 8.530332573 | 3.092601989 | 4.47E-06 | 0.000162656 | 1.982992072 | NEG | Up |
| 4-Fluoro-.alpha.-pyrrolidinobutiophenone | 8.429463158 | 3.075440754 | 0.012940926 | 0.044276565 | 1.08007045 | POS | Up |
| 6-Methyl-1H,6H,7H-pyrrolo[2,3-c]pyridin-7-one | 8.847889461 | 3.145333362 | 0.012146263 | 0.042304838 | 1.908917083 | POS | Up |
| 2-Hydroxy-2-methylbutyric acid | 5.089024162 | 2.347389041 | 0.008043092 | 0.033418652 | 1.070461087 | NEG | Up |
| 2-Carboxybenzeneboronic acid | 35.9495077 | 5.167900109 | 0.000507141 | 0.004350229 | 1.215641816 | NEG | Up |
| PI(16:1(9Z)/18:1(9Z)) | 41.02617424 | 5.358472722 | 0.00028099 | 0.002613575 | 1.569207179 | POS | Up |
| 4-Phenyl-1H-benzo[f]isoindole-1,3(2H)-dione | 7.965290777 | 2.993727029 | 0.00031643 | 0.002844322 | 1.822675789 | POS | Up |
| AS_1-5 | 4.911026134 | 2.2960245 | 0.018835849 | 0.058137451 | 1.215261265 | POS | Up |
| 4-(Dihydroxyboryl)benzoic acid | 20.64117037 | 4.36745287 | 0.000108733 | 0.001403063 | 1.504016006 | NEG | Up |
| 3-(2H-Tetraazol-5-yl)benzoic acid | 7.069101723 | 2.821526902 | 0.00081408 | 0.006088434 | 1.308502208 | NEG | Up |
| 2-[4-(Dibutylamino)-2-hydroxybenzoyl]benzoic acid | 2.373751087 | 1.247168661 | 0.00931058 | 0.03740369 | 1.95937718 | NEG | Up |
| (4R)-4-((1R,3S,5S,7R,9S,10S,13R,14S,17R)-1,3,7-trihydroxy-10,13-dimethylhexadecahydro-1H-cyclopenta[a]phenanthren-17-yl)pentanoic acid | 44.18199397 | 5.465386625 | 7.11E-06 | 0.000188876 | 1.686064922 | POS | Up |
| Allocholic acid | 26.62570903 | 4.734748039 | 4.71E-06 | 0.000168253 | 1.604164704 | NEG | Up |
| 2-Hydroxy-3-methylbutyric acid | 5.103051774 | 2.351360279 | 0.008058477 | 0.033435218 | 1.068000511 | NEG | Up |
| Myoseverin | 95.82082598 | 6.582267345 | 0.00016469 | 0.001765702 | 1.45522071 | POS | Up |
| PC(0:0/16:0)[U] | 4.90642841 | 2.29467321 | 0.019486727 | 0.059530415 | 1.072155957 | POS | Up |
| (4R)-4-((1R,3S,5S,7R,9S,10S,12S,13R,14S,17R)-1,3,7,12-tetrahydroxy-10,13-dimethylhexadecahydro-1H-cyclopenta[a]phenanthren-17-yl)pentanoic acid | 19.01389507 | 4.248982199 | 0.00038368 | 0.003290858 | 1.402936391 | POS | Up |
| 3-Hydroxy-2',4,4',6'-tetramethoxychalcone | 3.531076181 | 1.820107947 | 0.03498286 | 0.092592097 | 1.096555412 | POS | Up |
| PC(22:6(4Z,7Z,10Z,13Z,16Z,19Z)/18:2(9Z,12Z)) | 75118.76723 | 16.19688577 | 1.03E-07 | 1.29E-05 | 1.829238653 | POS | Up |
| 8-(2,3-Dihydroxy-3-methylbutoxy)-4-methoxy-1-methylquinolin-2(1H)-one | 5.82777509 | 2.542945201 | 0.007444066 | 0.029265755 | 1.711466318 | POS | Up |
| N-Arachidonoyl-L-serine | 520.4764931 | 9.023689197 | 1.34E-05 | 0.00029124 | 1.977606553 | POS | Up |
| Aigialomycin D | 9.174443977 | 3.197620725 | 0.008924594 | 0.033627344 | 1.019074564 | POS | Up |
| Zolpidem phenyl-4-carboxylic acid | 5.377676304 | 2.426982918 | 0.005882379 | 0.026439442 | 1.422955774 | NEG | Up |
| 3,3',5,5'-Tetraisopropylbiphenyl-4,4'-diol | 61.10663697 | 5.933257179 | 0.003807755 | 0.018082815 | 1.414061032 | POS | Up |
| DL-2,3-Diaminopropionic acid | 94.47118796 | 6.561802495 | 0.00133186 | 0.00838633 | 1.235806894 | POS | Up |
| Carnitine | 4.828982109 | 2.271719119 | 8.10E-06 | 0.00020503 | 2.013161019 | POS | Up |
| 2,2'-Bipyridine-4,4'-dicarboxylic acid | 2.595849528 | 1.376206758 | 0.019916616 | 0.065167642 | 1.475469606 | NEG | Up |
| Methanone, 1-naphthalenyl-(1-pentyl-1H-pyrrol-2-yl)- | 1.044431501 | 0.062717876 | 0.006026029 | 0.025003125 | 1.069593735 | POS | Up |
| (E)-1-Hydroxy-2-methylbut-2-enyl 4-diphosphate | 97.91584962 | 6.613470502 | 0.000408714 | 0.003665089 | 1.590237408 | NEG | Up |
| 2-Aminobutyric acid | 2.997987056 | 1.583994154 | 0.002226253 | 0.012084142 | 1.663745842 | POS | Up |
| 8'-Apocapsorbinal | 58.62404451 | 5.873420599 | 0.004442115 | 0.020077074 | 1.50027527 | POS | Up |
| Metalaxyl | 10.5800593 | 3.403275809 | 5.04E-05 | 0.00072434 | 1.526440096 | POS | Up |
| 26,27-Dinorcholest-5-en-24-yne-3,20-diol, (3.beta.)- | 31.63487314 | 4.983443905 | 0.010573817 | 0.038138833 | 1.362690242 | POS | Up |
| 1H-Indole-5-sulfonamide, N-(3-chlorophenyl)-3-[[3,5-dimethyl-4-[(4-methyl-1-piperazinyl)carbonyl]-1H-pyrrol-2-yl]methylene]-2,3-dihydro-N-methyl-2-oxo-, (3Z)- | 34.94048963 | 5.12682792 | 0.009852964 | 0.038797809 | 1.104904527 | NEG | Up |
| 2,6-Xylidine | 86.98500042 | 6.442694741 | 0.000737609 | 0.00530587 | 1.255004162 | POS | Up |
| Neogrifolin | 12.74546471 | 3.671912071 | 0.019547126 | 0.064454086 | 1.632627662 | NEG | Up |
| 2',6'-Dihydroxy-4-methoxychalcone-4'-O-neohesperidoside | 1.134303215 | 0.181806344 | 0.011652406 | 0.044089929 | 1.20914968 | NEG | Up |
| N-Cyclohexyl-N'-[2-(1H-imidazol-4-yl)ethyl]urea | 1.722478568 | 0.784486032 | 0.032840934 | 0.088109058 | 1.210311827 | POS | Up |
| Corchoroside_B | 19.90050065 | 4.314732821 | 0.026992144 | 0.075972878 | 1.035096182 | POS | Up |
| (22E,24R)-Stigmasta-4,22-diene-3,6-dione | 20.3375118 | 4.346071278 | 1.22E-05 | 0.00027107 | 1.574014693 | POS | Up |
| Neolinustatin | 5518.816843 | 12.43014329 | 3.68E-09 | 1.29E-06 | 2.14761331 | POS | Up |
| (2S,3R,4R,5R,6R)-2-Methyl-6-[[(2R,3S,4S,5R,6R)-3,4,5-trihydroxy-6-[(E)-7-hydroxy-3,7-dimethyloct-3-enoxy]oxan-2-yl]methoxy]oxane-3,4,5-triol | 7.170704455 | 2.842114857 | 0.019577296 | 0.064479566 | 1.685989395 | NEG | Up |
| 2-Mercaptobenzothiazole | 3.18753957 | 1.672443252 | 0.004656091 | 0.022275487 | 1.554108477 | NEG | Up |
| L-Linalool_3-[xylosyl-(1->6)-glucoside] | 227.3750456 | 7.828930117 | 2.14E-05 | 0.000396125 | 1.865991559 | POS | Up |
| [8-[2-(3-methylbutanoyloxy)propan-2-yl]-2-oxo-8,9-dihydrofuro[2,3-h]chromen-9-yl] (Z)-2-methylbut-2-enoate | 2.885468684 | 1.528805674 | 0.022098436 | 0.065687309 | 1.435673788 | POS | Up |
| Scrophularoside A8 | 7.91335199 | 2.984288931 | 0.030542239 | 0.089446291 | 1.419694018 | NEG | Up |
| Docosapentaenoic acid (DPA) | 8.500832044 | 3.087604056 | 0.018122685 | 0.060485973 | 1.119418067 | NEG | Up |
| Balletetroside | 78.26163516 | 6.290233348 | 0.00077048 | 0.005849899 | 1.476701869 | NEG | Up |
| Procyanidin B2 | 12.08749991 | 3.595443974 | 0.004759447 | 0.022671299 | 1.856322204 | NEG | Up |
| Thiomiltefosine | 15.96535862 | 3.996873054 | 0.00340946 | 0.016595097 | 1.44780879 | POS | Up |
| geraniol | 7.452278334 | 2.897681558 | 0.000372643 | 0.003225742 | 1.491591235 | POS | Up |
| (1S,3R,4S,5R)-4-{[(2E)-3-(3,4-dihydroxyphenyl)prop-2-enoyl]oxy}-1,3,5-trihydroxycyclohexane-1-carboxylic acid | 11.17510483 | 3.48221646 | 1.31E-05 | 0.000336156 | 2.43163954 | NEG | Up |
| 11b,13-Dihydrolactucopicrin | 3.807054859 | 1.928675359 | 0.048147233 | 0.125196471 | 1.54454922 | NEG | Up |
| 3'-Hydroxyflavanone | 4.381375326 | 2.131383807 | 0.038983298 | 0.107199194 | 1.036625892 | NEG | Up |
| 2,2-Dihydroperoxypropane | 25.0776364 | 4.648329473 | 0.014743424 | 0.048789031 | 1.381791763 | POS | Up |
| N-[2-(1H-Indol-3-yl)ethyl]nicotinamide | 7.266819619 | 2.861324095 | 0.003565429 | 0.017120153 | 1.097252658 | POS | Up |
| 3-Methyldioxyindole | 20.15408814 | 4.333000606 | 0.012970919 | 0.044325196 | 1.500174392 | POS | Up |
| Isonocardicin C | 17.83505812 | 4.156644012 | 0.026405253 | 0.08032633 | 1.358163724 | NEG | Up |
| Glycocholic acid | 37.75855544 | 5.238731666 | 0.002508837 | 0.014043868 | 1.07258975 | NEG | Up |
| 2-Ethyl-2-hydroxybutyric acid | 4.108983607 | 2.038781575 | 0.013039473 | 0.047689942 | 1.007562664 | NEG | Up |
| 2-Hydroxyhexanoic acid | 4.108060028 | 2.038457263 | 0.01306426 | 0.047717102 | 1.007265936 | NEG | Up |
| GlcCer(d18:1/16:0) | 4.529256382 | 2.179274206 | 0.037159512 | 0.09596864 | 1.496952266 | POS | Up |
| Glutamine | 4.613306873 | 2.205801262 | 0.00726293 | 0.028775134 | 1.296855906 | POS | Up |
| Allantoin | 13.5784508 | 3.763246983 | 0.010779773 | 0.041501689 | 1.125605332 | NEG | Up |
| Glyco-gamma-muricholic acid | 37.67148291 | 5.23540092 | 0.002518425 | 0.014058904 | 1.071474753 | NEG | Up |
| Cholic acid 7-sulfate | 7.589848124 | 2.924071017 | 0.028406427 | 0.084910347 | 1.717815909 | NEG | Up |
| 9-Oxooctadecanoic acid | 2.010303983 | 1.007413671 | 0.040344381 | 0.109843021 | 1.080843652 | NEG | Up |
| MES | 2.586017651 | 1.370732122 | 0.005728634 | 0.026016518 | 1.195503555 | NEG | Up |
| 7-Ketodeoxycholic acid | 12.7152507 | 3.668488002 | 0.046405286 | 0.121729851 | 1.601048252 | NEG | Up |
| Choline | 2.971252607 | 1.571071264 | 0.003965175 | 0.018560748 | 1.432120204 | POS | Up |
| omega-Muricholic acid | 26.62436355 | 4.734675133 | 4.74E-06 | 0.000168253 | 1.604417352 | NEG | Up |
| Hydroxyisocaproic acid | 4.108060028 | 2.038457263 | 0.01306426 | 0.047717102 | 1.007265936 | NEG | Up |
| 4-Hydroxyphenylpyruvic acid | 4.308165067 | 2.107073528 | 0.048864161 | 0.126224761 | 1.544267889 | NEG | Up |
| Glyco-beta-muricholic acid | 37.67148291 | 5.23540092 | 0.002518425 | 0.014058904 | 1.071474753 | NEG | Up |
| Tyramine | 7.399528274 | 2.887433301 | 0.032905695 | 0.088204329 | 1.066861043 | POS | Up |
| Hyocholic acid | 15.99457336 | 3.999510605 | 0.025426504 | 0.072826356 | 1.101323674 | POS | Up |
| Monomethyl phthalate | 4.301452266 | 2.104823828 | 0.048821808 | 0.126221792 | 1.544327027 | NEG | Up |
| Maltotriose | 12.30865759 | 3.621601522 | 0.029817272 | 0.082074197 | 1.024634169 | POS | Up |
| Palmitoylcarnitine (Car(16:0)) | 5.128996062 | 2.358676464 | 0.039533189 | 0.099908886 | 1.256939858 | POS | Up |
| N-Methyl-L-asparagine | 2.71763052 | 1.442349326 | 0.021217441 | 0.068594677 | 1.331014477 | NEG | Up |
| alpha-Muricholic acid | 26.54851234 | 4.730559116 | 4.83E-06 | 0.000168947 | 1.603263698 | NEG | Up |
| Pyrrolidine | 2.265596221 | 1.179890764 | 0.014522821 | 0.048277637 | 1.069719382 | POS | Up |
| Ala-Gly | 2.631751406 | 1.396023219 | 0.024707155 | 0.076429506 | 1.309411539 | NEG | Up |
| beta-Muricholic acid | 26.62436355 | 4.734675133 | 4.74E-06 | 0.000168253 | 1.604417352 | NEG | Up |
| Gly-Ala | 2.71763052 | 1.442349326 | 0.021217441 | 0.068594677 | 1.331014477 | NEG | Up |
| PC(18:0/16:0) | 11.26325714 | 3.493552185 | 0.025181267 | 0.072438983 | 1.199747198 | POS | Up |
| PC(17:0/17:0) | 11.25857107 | 3.492951828 | 0.025503608 | 0.072877884 | 1.198279811 | POS | Up |
| PC(16:0/18:0) | 11.26141008 | 3.493315578 | 0.025324107 | 0.072735599 | 1.199206601 | POS | Up |
| AG 1295 | 10.70806392 | 3.420625751 | 2.65E-05 | 0.000455933 | 1.948052639 | POS | Up |
| 2-Furoylglycine | 8.724257763 | 3.125032396 | 0.006080249 | 0.026981532 | 1.64502334 | NEG | Up |
| 2-Aminophenol | 11.8857791 | 3.571164569 | 8.38E-05 | 0.001044918 | 1.718052451 | POS | Up |
| (.+/-.)-Dropropizine | 8.458970448 | 3.080482082 | 0.003977694 | 0.018560748 | 1.100918357 | POS | Up |
| o-Anisidine | 17930.11575 | 14.13009718 | 1.33E-08 | 3.55E-06 | 2.081604309 | POS | Up |
| Soyacerebroside II | 3.058359927 | 1.612758202 | 0.027126986 | 0.07627598 | 1.193335175 | POS | Up |
| cis-7-Hexadecenoic acid | 1.720811484 | 0.783089058 | 0.018365291 | 0.061221348 | 1.391688952 | NEG | Up |
| Tripropylene glycol | 1.472924654 | 0.558683632 | 0.020997785 | 0.062881426 | 1.08067885 | POS | Up |
| 2-Acetylpyrrole | 26.2868392 | 4.716268774 | 8.69E-05 | 0.001070848 | 1.715451636 | POS | Up |
| 2-Carbamoylpyridine-3-carboxylic acid | 3.76101099 | 1.911120522 | 0.001783045 | 0.010915392 | 1.024252949 | NEG | Up |
| 3-Hydroxyoleylcarnitine | 14.21037864 | 3.828873091 | 0.016567539 | 0.053209485 | 1.481851248 | POS | Up |
| 1-O-(2,3,19,23-Tetrahydroxy-23,28-dioxours-12-en-28-yl)hexopyranose | 1.048175888 | 0.067880828 | 0.000138352 | 0.001656914 | 1.484191218 | NEG | Up |
| 4-Chloro-5,6,7,8-tetrahydroquinazolin-2-amine | 2.805240384 | 1.488124402 | 0.035978327 | 0.094099741 | 1.234962016 | POS | Up |
| (3-Nitrophenyl)methanamine | 2.417017265 | 1.273227779 | 0.011629093 | 0.040680102 | 1.515310404 | POS | Up |
| 3-Carboxy-4-methyl-5-propyl-2-furanpropanoic_acid | 5.402572852 | 2.433646621 | 0.019632866 | 0.064585199 | 1.024416715 | NEG | Up |
| Tsugaric acid A | 2264.479392 | 11.14496369 | 2.11E-08 | 4.40E-06 | 2.01807212 | POS | Up |
| 3.alpha.,7.alpha.-Dihydroxy-12-oxocholanoic acid | 13.58869554 | 3.764335064 | 1.98E-05 | 0.000439574 | 1.457038534 | NEG | Up |
| Laccarin | 27.62801488 | 4.788060001 | 3.60E-05 | 0.000581751 | 1.497195107 | POS | Up |
| 5,7,2'-Trihydroxyflavone | 92.70096285 | 6.534512419 | 0.001187478 | 0.007685773 | 1.386276107 | POS | Up |
| Oxohexadecadienoylcarnitine | 23.03172723 | 4.525550703 | 0.011493241 | 0.040305262 | 1.367743854 | POS | Up |
| Linoleoylcarnitine | 21.27425722 | 4.411036857 | 0.000396469 | 0.003364605 | 1.812076926 | POS | Up |
| Cholic acid | 15.96458514 | 3.996803158 | 0.025542783 | 0.072879307 | 1.10071895 | POS | Up |
| (3.beta.,5.Xi.,9.Xi.,18.alpha.)-3-Hydroxy-11-oxoolean-12-en-30-oic acid | 1.482361495 | 0.567897312 | 0.0044993 | 0.020254062 | 1.059045577 | POS | Up |
| Acetylcholine | 141.7046011 | 7.146742793 | 0.01742959 | 0.055264722 | 1.546433994 | POS | Up |
| Oxotetradecanoylcarnitine | 496.0362943 | 8.954301874 | 0.00046405 | 0.003785524 | 1.553507956 | POS | Up |
| Formylmethionine | 2.586017651 | 1.370732122 | 0.005728634 | 0.026016518 | 1.195503555 | NEG | Up |
| 3-Oxocholic acid | 12.7152507 | 3.668488002 | 0.046405286 | 0.121729851 | 1.601048252 | NEG | Up |
| 9Z,11E,13E-Octadecatrienoic acid | 1.903900002 | 0.928957706 | 0.003665032 | 0.017538458 | 1.078594579 | POS | Up |
| Diisooctyl phthalate | 1.331601363 | 0.413162252 | 0.004132133 | 0.01907504 | 1.003611886 | POS | Up |
| 2-Naphthalenecarboxamide, N-[2-(4-oxo-1-phenyl-1,3,8-triazaspiro[4.5]dec-8-yl)ethyl]- | 3.836779281 | 1.939895773 | 0.009360669 | 0.037510902 | 1.291741595 | NEG | Up |
| Tetraglyme | 1.802367133 | 0.84989291 | 0.011177877 | 0.039470343 | 1.525749095 | POS | Up |
| N2-Acetyl-N-(2-(4-hydroxyphenyl)ethyl)-.alpha.-glutamine | 2.365012938 | 1.241848076 | 0.023417403 | 0.073444869 | 1.457321533 | NEG | Up |
| 4-[1,2,4]Triazolo[3,4-b][1,3,4]thiadiazol-6-ylphenylamine | 60.32887271 | 5.91477672 | 0.035807674 | 0.093828132 | 1.040286603 | POS | Up |
| 6-Sialyllactose | 4.023956901 | 2.008614853 | 0.022024104 | 0.070183479 | 1.35409847 | NEG | Up |
| 1-Mercapto[1,2,4]triazolo[4,3-a]quinoxalin-4(5H)-one | 4.774463569 | 2.255338649 | 0.003919418 | 0.01969382 | 1.082200163 | NEG | Up |
