# Supplementary material for: Dysbiosis of gut microbiota and metabolomic alterations in myasthenia gravis: insights from 16S rRNA sequencing and untargeted metabolomics
Source: Front Immunol. 2026 Apr 23;17:1799199. doi: 10.3389/fimmu.2026.1799199 (PMC13149435; doi:10.3389/fimmu.2026.1799199)
Supplement: Supplementary file 6 [file Table6.docx]

Supplementary Table 5: Baseline Characteristic

| **Variables** | **Male,n=11** | **Female,n=18** | **Total,n=29** | **Statistical value** | ***P*** |
| --- | --- | --- | --- | --- | --- |
| Age (Year, Mean ± SD) | 53.64 ± 15.65 | 56.44 ± 13.21 | 55.38 ± 13.97 | t = -0.522 | 0.606 |
| QMG (Median, IQR) | 20.0 (18.0-21.0) | 17.5 (15.0-21.5) | 18.0 (15.0-21.0) | U = 128.0 | 0.199 |
| AChR (nmol/L, Median, IQR) | 7.22 (3.76-27.63) | 11.30 (0.98-26.18) | 8.69 (1.50-27.58) | U = 105.0 | 0.805 |
