# Supplementary material for: Dysbiosis of gut microbiota and metabolomic alterations in myasthenia gravis: insights from 16S rRNA sequencing and untargeted metabolomics
Source: Front Immunol. 2026 Apr 23;17:1799199. doi: 10.3389/fimmu.2026.1799199 (PMC13149435; doi:10.3389/fimmu.2026.1799199)
Supplement: Supplementary file 7 [file Table7.docx]

Differential metabolites in the MG and HC group( AUC >0.9)

| **No.** | **differential metabolites** | **AUC** |
| --- | --- | --- |
| 1 | Lutein5,6-epoxide | 1 |
| 2 | 1-Myristoyl-sn-glycero-3-phosphocholine (LPC(14:0/0:0) | 0,9921 |
| 3 | Pepstatin A | 0,9762 |
| 4 | Tetrafluorosuccinic acid | 0,9722 |
| 5 | 2-Ethylpyrazine | 0,9683 |
| 6 | 2,6-Dimethylpvrazine | 0,9683 |
| 7 | 2,5-Dimethylpyrazine | 0,9683 |
| 8 | Aminopicoline | 0,9683 |
| 9 | Glvcerophospho-N-palmitovlethanolamine | 0,9643 |
| 10 | Cinnamvlideneacetic acic | 0,9563 |
| 11 | 3-[(5,6-Diphenylfuro[2,3-d]pyrimidin-4-yl)amino]-1-propanol | 0,9563 |
| 12 | Methvl 1-piperazinecarboxvlate | 0,9524 |
| 13 | Xanthinol | 0,9524 |
| 14 | 4-(Trifluoromethyl)piperidine | 0,9484 |
| 15 | N-(1-Amino-3,3-dimethyl-1-oxobutan-2-yl)-1-pentyl-1H-indole-3-carboxamide | 0,9484 |
| 16 | (2E,6E,11E,13E)-18-(2,6-Dioxopiperidin-4-yl)-9-hydroxy-8-methoxy-10,12,14-trimethyl-15-oxooctadeca-2,6,11,13-tetraenoic acid | 0,9484 |
| 17 | 2-Decyl-3-hvdroxypentanedioic acid | 0,9405 |
| 18 | 2-Methylglutaric acid | 0,9365 |
| 19 | 3-Methylglutaric acid | 0,9365 |
| 20 | (1S,3R,4S,5R)-4-{[(2E)-3-(3,4-dihydroxyphenyl)prop-2-enoyl]oxy}-1,3,5-trihydroxycyclohexane-1-carboxylic acid | 0,9365 |
| 21 | 4-4-(Dimethylamino)methyl-2,5-dimethoxyphenyl-2-methyl-2,7-naphthyridin-1-one | 0,9365 |
| 22 | Adipicacid | 0,9365 |
| 23 | 4-Hvdroxy-4'-methyldiphenylamine | 0,9365 |
| 24 | 4-(2,2-Diphenvlacetvl)oxv\|-1,1-dimethvlpiperidiniumcatior | 0,9365 |
| 25 | 2,8,9-1rlisobutvl-2,5,8,9-tetraaza-1-phosphab1cvclol3,3,3lundecanesolutor | 0,9365 |
| 26 | Fluorofenidone | 0,9325 |
| 27 | Orotic acid | 0,9325 |
| 28 | Butanoic acid | 0,931 |
| 29 | LPC（13:0） | 0,9286 |
| 30 | LysoPC(14:1(9Z)) | 0,9286 |
| 31 | 1H-Indole-1-pentanoic acid, 3-[[[1-(aminocarbonyl)-2,2-dimethylpropyl]amino]carbonyl]- | 0,9286 |
| 32 | 3,12-Dihydroxy-13-methoxypodocarpa-8,11,13-trien-7-one | 0,9286 |
| 33 | YERAPAMII | 0,9246 |
| 34 | 4'-Hvdroxv-3'-methvlacetophenone | 0,9206 |
| 35 | 4-Allylcatecho | 0,9206 |
| 36 | 2'-Hydroxy-4'-methylacetophenone | 0,9206 |
| 37 | 2'-Hvdroxv-5'-methvlacetophenone | 0,9206 |
| 38 | 2,6-Dimethyl-4-hydroxybenzaldehyde | 0,9206 |
| 39 | Desoxypipradrol | 0,9206 |
| 40 | 3-Phenylpropanoic acid | 0,9206 |
| 41 | 4-oxododecanedioic acid | 0,9167 |
| 42 | N-Acetylmuramic acid | 0,9167 |
| 43 | 5-[1-(Phenylmethyl)-1H-indazol-3-yl]-2-furanmethanol | 0,9167 |
| 44 | [6]-Gingerdio]3,5-diacetate | 0,9127 |
| 45 | Cholest-4-en-3-one | 0,9127 |
| 46 | Carnitine | 0,9127 |
| 47 | N-Cyclohexylbenzenecarbothioamide | 0,9127 |
| 48 | Butalbital | 0,9087 |
| 49 | Benzamide,N-1H-pyrrolo2,3-cpyridin-5-yl | 0,9087 |
| 50 | Cryptotanshinone | 0,9087 |
| 51 | 3-Methylhistidine | 0,9087 |
| 52 | TRICARBALLYLIC ACID | 0,9087 |
| 53 | 2,2-Dimethvl-N-[3-(trifluoromethvl)phenvllpropanamide | 0,9048 |
| 54 | Pimonidazole | 0,9048 |
| 55 | 2-(Methylamino)-1-(morpholin-4-yl)ethan-1-one | 0,9048 |
| 56 | Cordycepin | 0,9048 |
| 57 | (2,5-Dioxotetrahydrofuran-3-yl)acetic acid | 0,9048 |
| 58 | Glycylproline | 0,9048 |
| 59 | Pyrrolidine,2-(diphenylmethyl)-,(2S) | 0,9008 |
